# Chemical Bonding: The Orthogonal Valence-Bond View 

**DOI:** 10.3390/ijms16048896

**Published:** 2015-04-21

**Authors:** Alexander F. Sax

**Affiliations:** Department of Chemistry, University of Graz, Heinrichstrasse 28, 8010 Graz, Austria; E-Mail: alexander.sax@uni-graz.at; Tel.: 43-316-380-5513, Fax: 43-316-380-9850

**Keywords:** bonding, bond, theoretical entity, VB, OVB, interference, VB reading, diabatic processes

## Abstract

Chemical bonding is the stabilization of a molecular system by charge- and spin-reorganization processes in chemical reactions. These processes are said to be local, because the number of atoms involved is very small. With multi-configurational self-consistent field (MCSCF) wave functions, these processes can be calculated, but the local information is hidden by the delocalized molecular orbitals (MO) used to construct the wave functions. The transformation of such wave functions into valence bond (VB) wave functions, which are based on localized orbitals, reveals the hidden information; this transformation is called a VB reading of MCSCF wave functions. The two-electron VB wave functions describing the Lewis electron pair that connects two atoms are frequently called covalent or neutral, suggesting that these wave functions describe an electronic situation where two electrons are never located at the same atom; such electronic situations and the wave functions describing them are called ionic. When the distance between two atoms decreases, however, every covalent VB wave function composed of non-orthogonal atomic orbitals changes its character from neutral to ionic. However, this change in the character of conventional VB wave functions is hidden by its mathematical form. Orthogonal VB wave functions composed of orthonormalized orbitals never change their character. When localized fragment orbitals are used instead of atomic orbitals, one can decide which local information is revealed and which remains hidden. In this paper, we analyze four chemical reactions by transforming the MCSCF wave functions into orthogonal VB wave functions; we show how the reactions are influenced by changing the atoms involved or by changing their local symmetry. Using orthogonal instead of non-orthogonal orbitals is not just a technical issue; it also changes the interpretation, revealing the properties of wave functions that remain otherwise undetected.

## 1. Introduction

### 1.1. Bonding and Bonds

In a composite system composed of two or more subsystems, the subsystems do not initially interact if they are spatially well separated. In a chemical context, the system is often a molecule, and the subsystems will be called fragments. If the distance between the subsystems is reduced, then interactions between the subsystems can be recognized; e.g., by the forces attracting or repelling the subsystems. In the majority of cases, attraction of the subsystems is accompanied by the release of energy in the form of heat; when the subsystems repel each other, the system has to consume energy to overcome the repulsion. The system’s state of minimum energy is called the equilibrium state; it is well characterized by properties, such as equilibrium geometry, equilibrium dipole moment, *etc*. Both stabilization and destabilization of the composite system are defined relative to the state of non-interacting subsystems, which one might call the canonical reference state.

We say that the attractive interaction between the subsystems stabilizes the system and that a repulsive interaction destabilizes the system. In some scientific disciplines, like chemistry, the process of stabilizing a composed system is called bonding, sometimes also binding. The stabilization energy, or bonding energy, is the best system property for creating a scale for the degree of stabilization; with such a scale, one can classify the stabilization as strong or weak and say that the strength of bonding is high or low, respectively. The initial system, composed of non-interacting subsystems, is called non-bonded, while the stabilized system is called bonded. Frequently, one speaks about the bonded system as if something causing the stabilization had been added to, or had appeared in, the system. This something is mostly called a bond.

When a system changes from non-bonded to bonded, not only the energy, but many more system properties will also change, and many of these changes can be experimentally monitored. Released energy can be measured; changes in the spatial distances can be detected with spectroscopic methods; while bonding in macroscopic systems can change material properties, such as ductility, elastic stiffness, plasticity, strain, strength, toughness, viscosity, and many more.

Changes in system properties are always reported relative to a reference state, which need not always be the canonical one; different reference states can be defined with varying degrees of physical plausibility. For example, if the increase in electron density between two atoms is used as the measure of bonding in a molecule and if the reference state is the canonical one, the change in the electron density is conceptually plausible: During the approach of the atoms, the atom densities change gradually due to mutual perturbation until, at the equilibrium geometry, the final molecular electron density is found. However, how should one compare electron densities at different system geometries? A possible, but physically implausible, reference state is created by summing the electron densities of non-interacting (free) atoms, each at its own position in the system’s equilibrium geometry. We know that when two atoms approach each other, the electron densities gradually change due to contraction and polarization, but the implausible reference state is constructed as if the completely separated atoms can be brought to the equilibrium distance, where they instantly change from the electron densities of the free atoms to the molecular electron density. In this model reaction, the interaction between the free atoms can be simply switched on at a certain geometry. Subtracting the electron density of the molecule from the reference density gives the electron-density difference, which is frequently used to show where electron density is accumulated between atoms, indicating a “bond” between the atoms, and where it is depleted, in which regions no bond can exist.

The use of words like “exist” indicates that we are talking about philosophical questions concerning the reality of things. The philosophical discipline in which such questions are discussed is called ontology. In the philosophy of science, the existence of entities (things, objects) and theories is frequently discussed on the basis of two antagonistic positions: Realism and anti-realism. Those who claim that entities do exist independently of a human observer, which means they claim that entities are real, are entity-realists; those who deny this claim are entity-anti-realists. Those who agree with the claim that theories are not a mere human construction, but exist, just as horses or cars do, are theory-realists, and those who claim that theories are only human constructs made to explain experiments or to make predictions are theory-anti-realists. Of course, this is an extremely crude sketch of these ontological positions, but it is sufficient to show that the spectrum of what can be claimed to exist is very broad. (Some books on the philosophy of science, where such topics are discussed, include those by Nancy Cartwright [1], Ian Hacking [2] and Ronald Giere [3,4]. These books are also understandable for natural scientists interested in philosophical issues.) Theories are necessary to explain or predict experimental outcomes, and they help to bring structure to an otherwise chaotic set of unrelated experimental facts. Scientific theories are sets of sentences formulated such that the relationship between two different entities in systems are described: those with which one can make experiments and those that are just claimed to exist. If a theory contains entities that are claimed to exist, although it is not possible to make experiments with them, and if such a theory is successful, then these entities are essential for its success. Such fictitious entities are called theoretical entities. According to Ian Hacking [2], one criterion to distinguish real entities from theoretical entities is the ability to manipulate them, *i.e.*, do experiments with them. It was criticized that this is too restrictive a criterion because scientists in disciplines like astronomy or cosmology observe their entities, but do not manipulate them. (An overview of critiques of Hacking’s entity realism can be found, e.g., in [5].) I propose that whether the properties of entities can be observed in a reproducible way should also be a criterion to distinguish theoretical entities from real entities. If it turns out that one can do experiments with theoretical entities or that one can make observations that can be attributed only to them, the status of the entity turns from theoretical to real.

Now, one could argue that non-real entities have no place in scientific theories because, after all, such theories claim to describe the “reality” or the “nature” of things, and this is not fictitious. However, scientific theories and especially theories of chemical bonding are full of theoretical entities, such as covalent bonds, multiple bonds, polar bonds, and so on. Gernot Frenking [6] called them the “unicorns in the world of chemical bonding models”. Other theoretical entities well known to molecular physics include the reduced mass and center of mass of a two-particle system or the different quasiparticles in many-body physics [5,7].

Therefore, why is speaking about a bond much more difficult than speaking about bonding? Most words used in scientific theories are taken from everyday language, just think of “force”, “velocity” or “bond”. Every word has its own conceptual history; the semantics of the word “bond” in everyday language is connection via a cord, rope, band or ligament; in general, a material joint. Using the word “bond” has the connotation of something that is localized in space, that is strong, more or less rigid and that binds, fastens, confines or holds together. This connotation is supported in chemistry by rendering techniques used in molecular modeling software, like ball and cylinder or ribbon. If this view of a bond is adopted, one can claim that bonds between atoms exist or do not exist, that they can be broken or unbroken, that one can make bonds where no bonds are or split existing bonds. Forming a new bond causes stabilization of the system, whereas splitting a bond causes destabilization. Covalent bonds possess directional preferences that are made responsible for the three-dimensional structure of many molecules; the origin of this anisotropy is frequently attributed to other theoretical entities called hybrid orbitals. The semantics of the words “bond” and “bonding” are actually very different; the latter is just used to describe the system stabilization due to interactions between subsystems.

Bond-making is frequently used synonymously with bonding to indicate system stabilization. On the other hand, there is no well-accepted word in chemistry that describes system destabilization and which can be used as an antonym for bonding; instead, one speaks about bond breaking or bond dissociation, but this immediately suggests the existence of a bond. I shall use the word “debonding” to describe the destabilization of a composite system; the word “bond” will only be used to describe elements in a structural formula, e.g., a C–H bond or a C=C double bond.

## 2. Covalent Bonding and Chemical Reactions

Bonding energies in chemistry range from a few kilojoules (weak hydrogen bonding) [8] to several hundreds of kilojoules (strong bonding); the physical origin of the differences in strength are the different interactions causing the stabilization. It has been shown that all weak bonding is caused by four basic interactions [9]: The interaction between static electric multipoles higher than monopoles in the different subsystems can be attractive, as well as repulsive; the interaction between static multipoles in one subsystem and multipoles induced in another are always attractive, as are the interactions between instantaneously created multipoles in one and induced multipoles in another subsystem. These three interactions are called electrostatics, induction and dispersion, respectively. Repulsive interactions are between the nuclei, but more important is that, for many electron atoms, electrons with like spin avoid being spatially close (Pauli principle). The result is the same as if strong repulsive forces, called Pauli forces, kept these electrons far apart. Depending on how large the contributions of the basis interactions are, weak bonding is called van der Waals bonding, hydrogen bonding, and so on [10]. Weak bonding, however, is not the topic of this paper.

Strong bonding covers ionic bonding, metallic bonding and covalent bonding. Ionic bonding is caused by long-range Coulomb interactions between ions (monopoles); these interactions are completely isotropic. Cations are formed when some atoms lose one or more valence electrons, whereas anions are formed when these electrons are strongly attracted by neutral atoms. When an electron changes from one site to another, one speaks of charge transfer. Although Coulomb interactions are isotropic, the result of ionic bonding is crystal grids, where isotropy is strongly reduced. In metallic bonding, atoms lose some of their valence electrons, and the cations then form a grid, similar to the case of ionic bonding. In contrast to ionic bonding, however, there are no atoms that can bind these electrons; instead, they are distributed among the cations in the grid. The metal crystal is stabilized by interactions between delocalized electrons and the cation grid. The characteristics of covalent bonding are a pronounced spatial anisotropy, a strong dependence on the electron spin and attractive interactions that are too strong and too short-range to be caused by those interactions that are responsible for weak bonding. When chemists talk about chemical bonding, most of the time they mean covalent bonding. Covalent bonding is the topic of this paper.

Chemical bonding and debonding occur in chemical reactions; complex reactions are considered to be composed of several elementary reactions, which are each named according to what happens between the reacting fragments: Dissociation, recombination, insertion, addition, elimination, substitution, and so on. In every elementary reaction, only a few atoms are involved—usually two and seldom more than three–and this is the reason why Levine simply stated: “Chemistry is local” [11]. Each elementary reaction may itself comprise several processes, such as spin-coupling and -decoupling, spin flip, local electron excitations in fragments and charge transfer between them. These are local processes, and they have a strong influence on the properties of the fragments, especially their geometries. Depending on the theoretical method used, the description of local processes may be easy, but can be very difficult. In classical valence bond (VB) theory, for example, the stabilization of two radical fragments in their respective doublet ground states by spin coupling to a singlet ground state (the formation of a Lewis electron pair) is well described by a single Heitler–London-type wave function. Such wave functions clearly show, by construction, the local character of the spin coupling; the delocalized molecular orbitals used in molecular orbital (MO) theory, on the other hand, hide this local process completely. Similarly, if the chemical environment of one of the atoms involved in an elementary reaction is changed, e.g., by substitution, the reactivity can change dramatically. Therefore, the question arises as to what is possible to explain what is responsible for such changes, in terms of the local processes. The use of terms like the “local Hund’s rule”, for instance, indicates that the importance of such local processes is known, but they are seldom revealed by the quantum chemical methods used to describe them (I am only referring to wave function methods; density functional methods are even worse in this respect).

All VB wave functions are, in general, linear combinations of configuration state functions (CSF) [12], based on orbitals describing the fragments. In conventional VB, or Slater–Pauling VB, the fragments are atoms and the orbitals are non-orthogonal atomic orbitals or hybrid orbitals; in non-conventional VB methods, the orbitals are either atom-centered atomic orbitals (AO) or basis functions, once Slater, now usually Gaussian, with small delocalization tails on other atoms, or the fragments are larger moieties than atoms, or both. The orbitals describing the fragments may be orthogonal or non-orthogonal. We speak of an orthogonal-valence-bond (OVB) method whenever the wave function is based on orthogonal fragment orbitals.

The multi-configurational self-consistent field (MCSCF) [12] is the most efficient wave-function method for describing chemical reactions; the CSFs are made with delocalized MOs. The best MCSCF wave functions are of the complete active space (CAS)-type [13,14], also called fully-optimized reaction space (FORS)-type [15,16,17,18,19,20,21], the latter description indicating that the wave function is designed to optimally describe the local processes during chemical reactions. However, the use of delocalized molecular orbitals hides the local processes occurring in chemical reactions; analysis of a FORS wave function is necessary to reveal them.

## 3. OVB Reading of FORS Wave Functions

The typical reactions of two or more fragments are recombination, insertion or addition reactions and the corresponding reverse reactions. The FORS wave function describing such reactions is a linear combination of CSFs made with orthogonal delocalized molecular orbitals (MO); it is completely determined by the system’s spin state and its spatial symmetry, the number *n*_MO_ and symmetry of the partially occupied MOs (the active orbitals) and the number of electrons *n*_elec_ that can be distributed among them (the active electrons). The information concerning the number of active electrons and active orbitals is abbreviated as CAS(*n*_elec_, *n*_MO_). The fragments are the reactants that are combined to give the product, while the definition of fragments is determined by the idea of how an elementary reaction proceeds and which MOs are needed to describe the reaction. If the choice of the active MOs and of the active electrons is correct, the FORS wave function will correctly describe static electron correlation during the reaction, although a quantitatively correct description needs to include dynamic correlation corrections. That means FORS wave functions do correctly describe the geometries of reactants, products and possible transition structures, but FORS energy differences between them are always too small [12]. The description of all reactions discussed in this paper is based on molecular structures where for fixed inter-fragment distances *R*, all fragment geometries are fully optimized.

Use of localized orbitals helps to reveal the local processes occurring during the reaction; if localized orbitals are obtained by separate orthogonal transformations in the space of active MOs and of doubly-occupied MOs, the electron density and the total energy of the molecule remain unchanged. A localization procedure using an orthogonal Procrustes transformation was recently described [22] that allows transformation from delocalized FORS-MOs to orthogonal fragment orbitals (FO). Transformation of the doubly-occupied MOs yields the same number of doubly-occupied FOs, and transformation of the *n*_MO_ active MOs yields *n*_MO_ FOs. When these FOs are used as active orbitals, the CSFs made with the FOs will describe the local processes during the reaction. Since the FOs are orthogonal, so are the CSFs made with them. The FO-CSFs describe which local states fragments are in, how these states are coupled and whether the fragments are neutral or ionic. A single diagonalization of the CI -matrix (*vide infra*) that was constructed with the FO-CSFs yields the energies and the weights of the FO-CSFs in the molecular state function. This analysis, which can be called an OVB reading of a FORS wave function, differs significantly in some aspects from a VB reading based on non-orthogonal orbitals.

In this paper, the OVB analysis of four reactions will be presented, each describing bonding in a molecular system composed of two subsystems. All four reactions involve four active electrons in four active MOs, leading to a CAS(4,4) singlet wave function. The reactions are:

2CH_2_ → C_2_H_4_

2SiH_2_ → Si_2_H_4_

CH_2_ + H_2_ → CH_4_

The dimerization reaction of two carbenes was investigated in *D*_2*h*_ symmetry, while the dimerization reaction of two silylenes was studied in *D*_2*h*_ and *C*_2*h*_ symmetry. Insertion of a carbene into the H–H single bond was studied in *C*_*s*_ symmetry.

**Figure 1:**
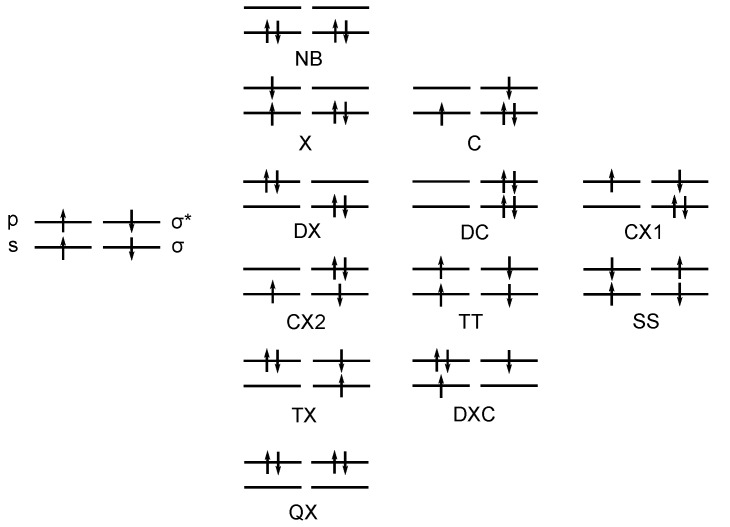
The types of fragment orbital (FO)-configuration state functions (CSFs) used for the insertion reaction. NB, the no-bond configuration, in which the lowest FO of each fragment is doubly occupied; X, a single excitation in one fragment; C, single-charge transfer; DX, double excitations in one fragment; DC, double-charge transfer; CX, single-charge transfer and single excitation; TT, singlet coupling of two local triplets; SS, singlet coupling of two local singlets; TX, double excitation in one fragment and a single excitation in the other; DXC, double excitation and a charge transfer; QX,quadruple excitation; *σ* and *σ*^∗^ are the bonding and antibonding H_2_ MOs, respectively.

For the three dimerization reactions, the four active orbitals of the product are the bonding and antibonding MOs of the double bond; for the insertion reaction, two bonding and the corresponding antibonding orbitals for two CH bonds of the methane molecule are the active orbitals. For the dimerization reactions, the FOs are the s and p lone pair orbitals of the carbene and silylene fragments; for the insertion reaction, the s and p lone pair orbitals of the carbene and the bonding and antibonding *σ* orbitals in the hydrogen molecule are the fragment orbitals. The types of CSFs that can be made with the FOs are listed in Figure 1.

According to the distribution of the four active electrons on the two fragments, the FO-CSFs can be classified as ionic or neutral (non-ionic). All CSFs with a C in the abbreviation are ionic CSFs; all others are neutral. For representing the results, only those CSFs with a weight larger than 0.1 somewhere along the reaction coordinate are included in the discussion; the number of these important CSFs varies with the system.

### 3.1. The Carbene Dimerization

The dimerization of two carbene fragments to form ethene was performed in *D*_2*h*_ symmetry using the 6-31G* basis. The order of the active MOs is σ, π, π^∗^ and σ^∗^. Of the 20 MO-CSFs, only 12 are of the *A*_*g*_ symmetry, and the ethene ground state of *A*_*g*_ symmetry was decomposed in these 12 MO-CSFs.

**Figure 2:**
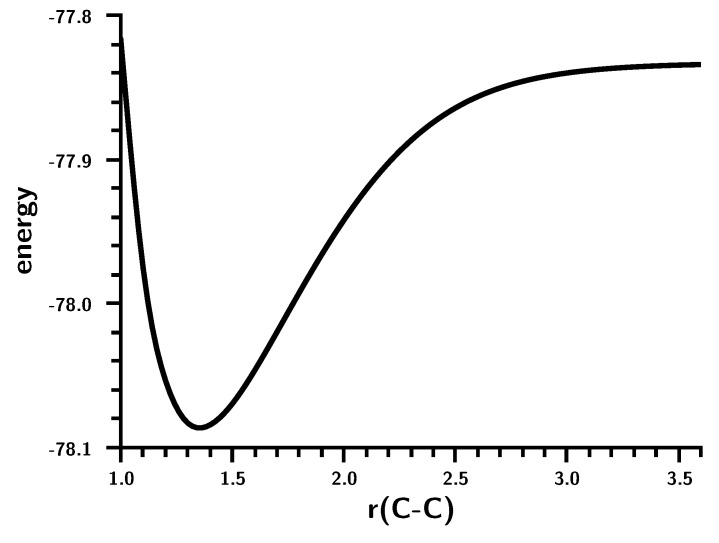
Carbene dimerization. The energy curve for the C–C bond stretching. Energy in Hartrees; bond length in Å.

The energy curve for the dimerization (Figure 2) shows a monotonic energy decrease from the non-interacting carbenes at large distances to the equilibrium geometry; at C–C distances larger than 2.6 Å, the decrease is rather slow; at smaller C–C distances, the system stabilization becomes stronger.

**Figure 3:**
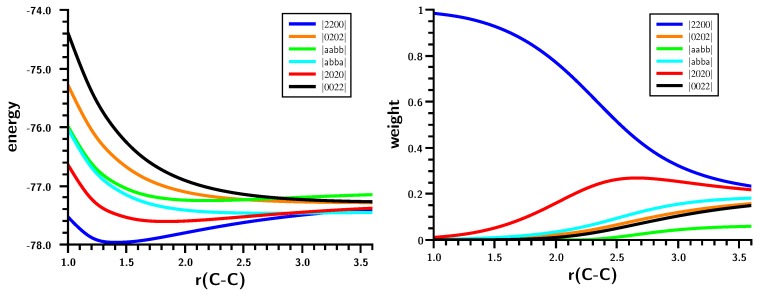
Carbene dimerization. Energy (**left**) and weights (**right**) of the six most important molecular orbital (MO)-CSFs. Energy in Hartrees; bond length in Å.

The distance dependence of the weights of the MO-CSFs (Figure 3) leads one to assume that the carbene dimerization occurs in a completely monotonic way. The Hartree–Fock determinant (CSF |2200| = |σ^2^π^2^|) has the highest weight; at equilibrium geometry, it is close to one. It decreases with increasing C–C distance, and other CSFs, like |2020| = |σ^2^π^∗2^|, then become important. At r(C–C) = 3.5 Å, the wave function is dominated by five CSFs with weights ≈ 0.2.

**Figure 4:**
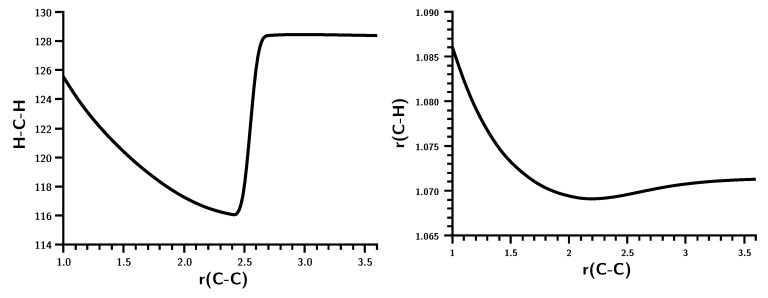
Carbene dimerization. HCH bond angle and CH bond length. Angles in degrees; bond lengths in Å.

Fragment properties (Figure 4), like the HCH bond angle, indicate a drastic and sudden change in the electronic structure of the fragments at the point where bonding becomes strong: The bond angle drops from about 128 degrees, which is slightly smaller than the typical HCH angle in triplet carbene (134.0 degrees), to 116 degrees and then increases to the final HCH angle in ethene. The C–H bond length at large C–C distances is typical for triplet carbene (1.077 Å) [23], shrinking slightly when bonding starts and then increasing to its final value.

**Figure 5:**
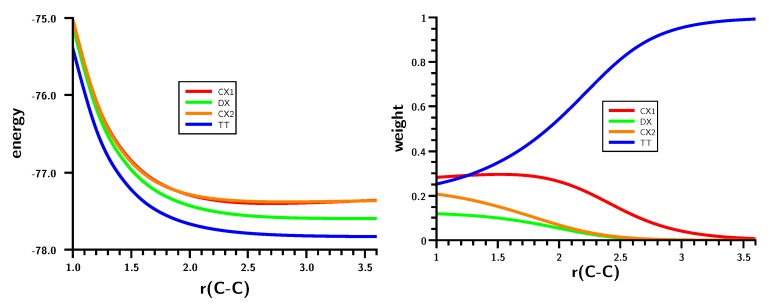
Carbene dimerization. Energies (**left**) and weights (**right**) of the six most important FO-CSFs. Energy in Hartrees; bond length in Å.

The OVB analysis (Figure 5) reveals what happens during the carbene dimerization: At large C–C distances, the wave function is dominated by the TT CSF, representing two triplet carbenes coupled to a singlet.

At r(C–C) = 2.6 Å, the single-charge transfer and single excitation CX1 CSF becomes important; its weight increases from zero to about 15%, while the weight of the TT CSF drops to 85%. With respect to the TT configuration, the ionic CSF describes a simple charge transfer from the s lone pair orbital at one fragment to the s lone pair orbital at the other. At the equilibrium C–C distance, both CSFs have equal weight of about 30%, and the weight of the CX2 CSF increases to nearly 20%. This ionic CSF describes the charge transfer from the p lone pair orbital at one fragment to the p lone pair orbital at the other. CX1 and CX2 have nearly identical energies, but their weights are significantly different. For none of the important FO-CSFs can a pronounced local minimum be found; the TT CSF is repulsive, while the ionic CSFs have nearly identical energy curves with an extremely shallow minimum around r(C–C) = 2.5 Å. Therefore, no single FO-CSF causes the deep minimum in the ground state; rather, the increasing weight of the ionic CSFs and the reduction of the weight of the non-ionic TT CSF are both responsible.

### 3.2. The Silylene Dimerization in D_2h_

**Figure 6:**
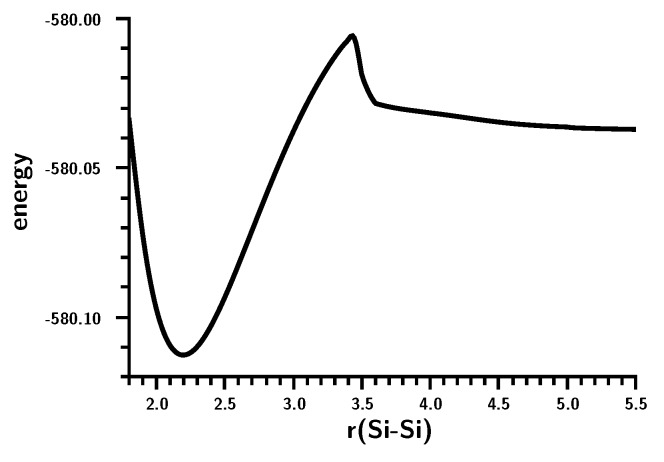
Planar silylene dimerization. The energy curve for the Si–Si bond stretching. Energy in Hartrees; bond length in Å.

**Figure 7:**
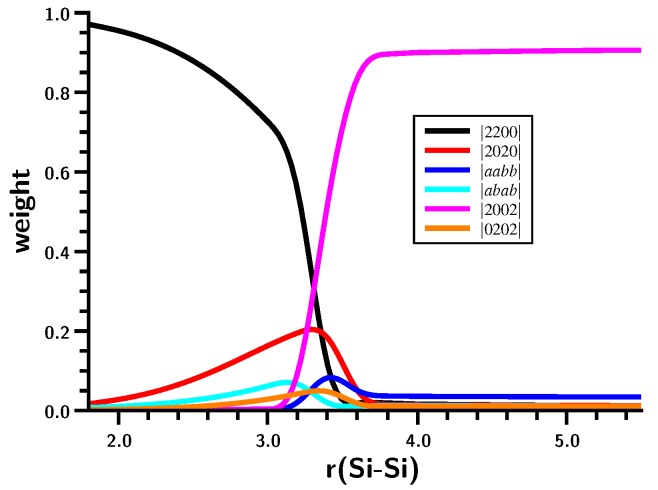
Planar silylene dimerization. Weights of the six most important MO-CSFs. Bond length in Å.

The energy curve (Figure 6) shows a local maximum caused by a drastic change in the wave function at an Si–Si distance of about 3.5 Å. At shorter distances, the Hartree–Fock determinant (CSF |2200| = |σ^2^*π*^2^|) dominates as in the case of ethene; on stretching the Si–Si bond, the weight of the |2020| = |σ^2^*π*^∗2^| CSF increases, but at 3.5 Å, the weight of both CSFs drops to zero and the |2002| = |σ^2^σ^∗2^| CSF reaches a weight of more than 90% within few tens of an Ångstrom (Figure 7). Interpretation of the change of character of the wave function on the basis of MOs is impossible; after all, the change occurs at an Si–Si distance that is more than 50% larger than the equilibrium distance, so the meaning of bonding and antibonding σ and π orbitals is far from clear. The maximum in the total energy curve suggests that the MO switch π ↔ σ^∗^ does not occur smoothly. Using a larger basis set is no remedy for this problem.

**Figure 8:**
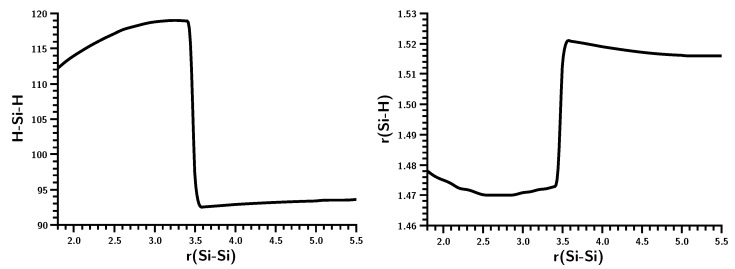
Planar silylene dimerization. HSiH bond angle (degrees) and SiH bond length (Å).

**Figure 9:**
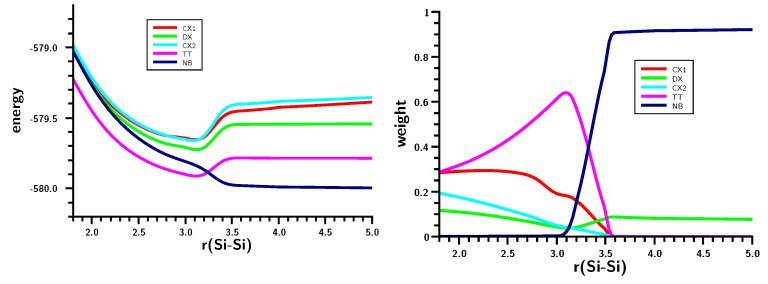
Planar silylene dimerization. Energies **(left)** and weight **(right)** of the six most important FO-CSFs. Energies in Hartrees; bond lengths in Å.

The geometry data of the silylene fragments (Figure 8) already suggest what happens at r(Si–Si) = 3.5 Å. For larger Si–Si distances, the HSiH bond angle has a typical value of the singlet ground state, and Si–H has a typical distance of about 1.52 Å. The experimental values are 1.516 Å and 92.8 degrees [24]. At 3.5 Å, the HSiH angle increases almost immediately to about 118 degrees, while the Si–H distance shrinks to less than 1.48 Å; both are close to typical values for triplet silylene (1.48 Å and 118.5 degrees) [25]. This suggests local singlet-triplet excitation in each fragment, and the weights of the FO-CSFs support this interpretation (Figure 9).

At large Si–Si distances, the wave function is dominated by NB (no-bond), representing the silylenes in their corresponding singlet ground states with a doubly-occupied s AO; the weight of this configuration is greater than 90%. The weight of double excitation (DX) is about 10% at large distances; this CSF describes the angular correlation in the singlet ground state by excitation of the lone pair electrons from the s to the p lone pair AO. Between r(Si–Si) = 3.5 and r(Si–Si) = 3.0 Å, NB disappears and is replaced mainly by TT and CX1; at r(Si–Si) = 3.0 Å, the weight of TT is greater than 60%, and the weight of the ionic CX1 is about 20%. The local excitations initiate bonding between the silylenes. With decreasing 1 Si–Si distance, the weight of TT decreases to about 30%, and the weight of the ionic CX1 increases to the same value. Together with CX2 (20%), the ionic contributions dominate the disilene wave function at the equilibrium geometry.

The shape of the FO-CSF energy curves (Figure 9) seems rather strange at first. However, it is well known that the electron distribution in high-spin systems is, due to Fermi correlation, more compact than in low-spin systems; accordingly, the silylene lone pair orbitals will be more compact after the triplet excitation than in the singlet ground state, and this contraction of the electron density seems to be energetically favorable for nearly all FO-CSFs. Only for NB with doubly-occupied s AOs are the compact s AOs unfavorable. The kinks in the energy curves might be responsible for the faster decrease in the total energy compared with the more moderate decrease found for the carbene dimerization.

### 3.3. The Silylene Dimerization in C_2h_

**Figure 10:**
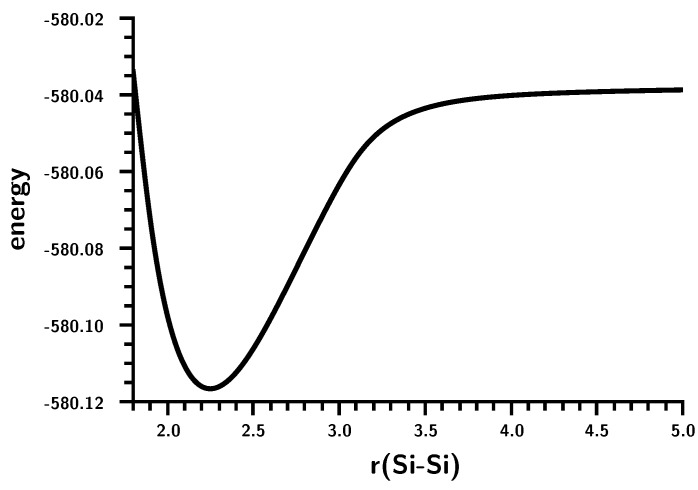
Non-planar silylene dimerization. The energy curve for the Si–Si bond stretching. Energy in Hartrees; bond length in Å.

**Figure 11:**
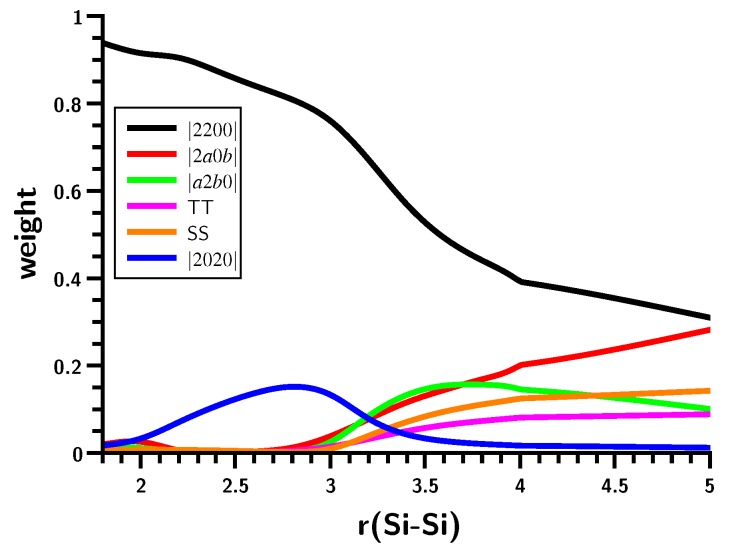
Non-planar silylene dimerization. Weights of the six most important MO-CSFs. Bond lengths in Å.

**Figure 12:**
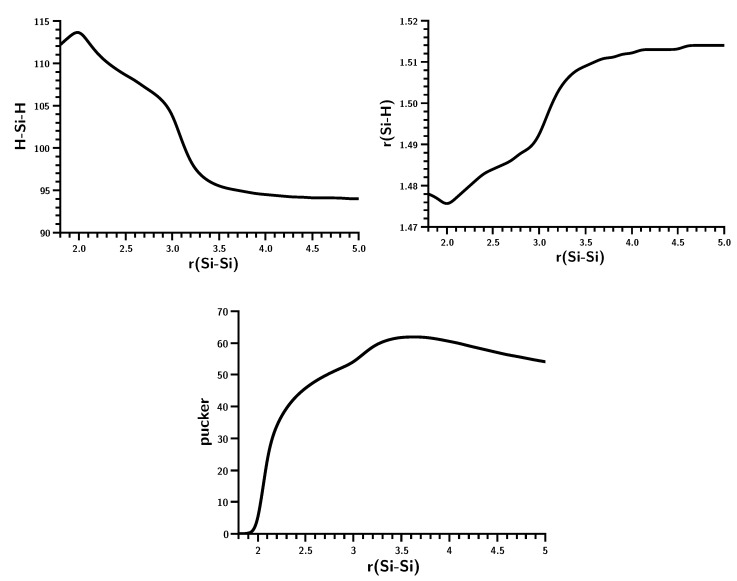
Non-planar silylene dimerization. HSiH bond angle, SiH bond length and pucker angle. Angle in degrees; bond length in Å.

There is no hump in the energy curve of the ground state (Figure 10); it more resembles the carbene dimerization than the planar silylene dimerization, but again, bonding occurs in a much smaller interval of the Si–Si distances; similar to that for planar dimerization. That the non-planar and planar dimerizations of silylenes are very different can be seen from the weights of the MO-CSFs (Figure 11). The Hartree–Fock determinant has the highest weight at short Si–Si distances; it decreases monotonically to about 30% at r(Si–Si) = 3.5 Å. At the same time, the weight of other CSFs increases. In contrast to the planar silylene dimerization, there is no switching of MO-CSFs, because, due to switching of MOs, due to the strong puckering of the silylene moieties, there is no σ − π separation, and the four active MOs can change their character smoothly. At very short Si–Si distances, disilene becomes planar, but even at the equilibrium geometry, disilene is puckered; the pucker angle is largest at r(Si–Si) = 3.5 Å where bonding starts (Figure 12).

**Figure 13:**
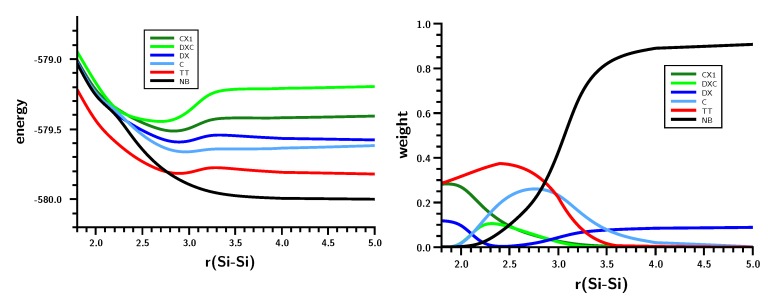
Non-planar silylene dimerization. Energies (**left**) and weights (**right**) of the six most important FO-CSFs. Energy in Hartrees; bond length in Å.

Furthermore, the changes in the HSiH bond angle and the Si–H bond length for the planar and non-planar dimerizations are very different. Even though the curves for non-planar dimerization are rather wiggly, both geometry parameters change less abruptly than for planar dimerization. The OVB analysis (Figure 13) shows that there is indeed a significant difference in the bonding processes. At larger distances, NB and the correlating DX are dominant, but when the Si–Si distance decreases, the single-charge-transfer CSF, C, becomes important, even ahead of TT. At shorter distances, C disappears and is replaced by CX1, describing charge-transfer coupled with local excitation. It is also noteworthy that the weight of NB is only zero when disilene becomes planar.

The shapes of the CSF energy curves indicate that the electron density at the silicon atoms contracts at r(Si–Si) = 3.5 Å due to the triplet excitation, although the energy jump is less pronounced than during planar dimerization. Nevertheless, the rapid energy decrease is again enhanced by contraction of the electron density in the local triplet states.

### 3.4. The Insertion of Carbene into H_2_

**Figure 14:**
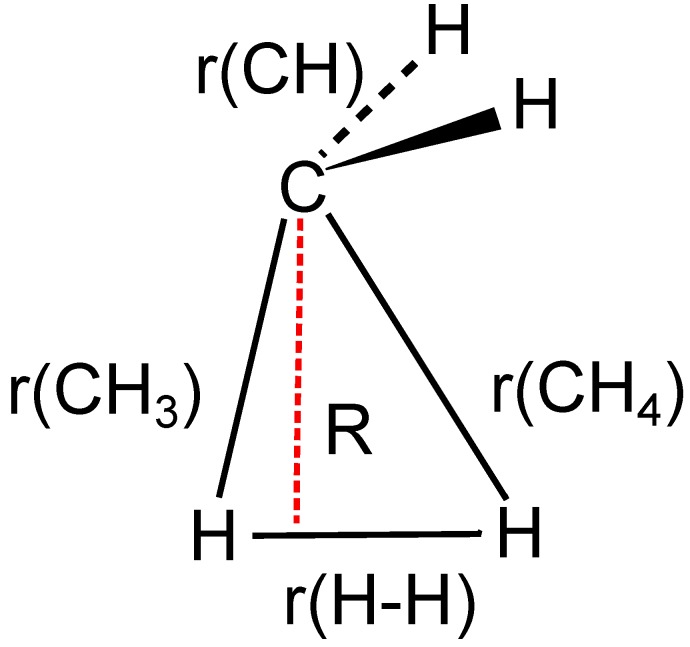
Carbene insertion into H_2_. Definition of the geometry parameters.

**Figure 15:**
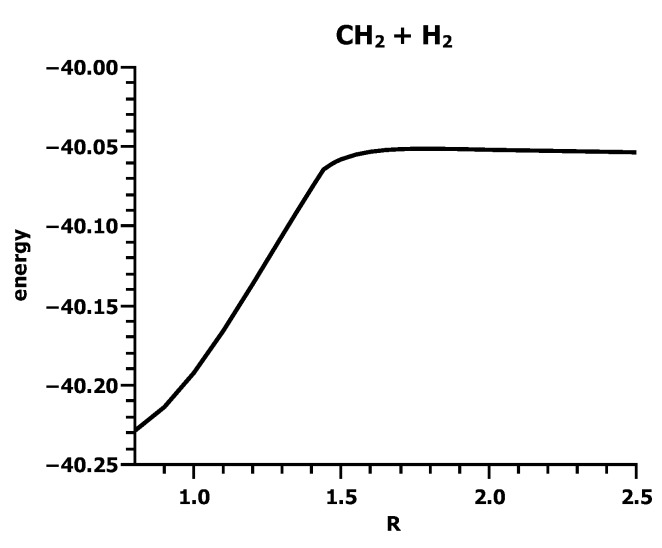
Carbene insertion into H_2_. The energy curve. Energy in Hartrees; *R* in Å.

This reaction was investigated in *C*_*s*_ symmetry. For all 20 CSFs, the coefficient is different from zero for symmetry reasons. The parameter *R* used as the reaction coordinate (Figure 14) is the normal distance of the carbon atom from the molecular axis in H_2_. The energy curve of the ground state (Figure 15) is as unspectacular as the energy curves for the carbene dimerization. According to the energy curve, bonding starts at about *R* = 1.5 Å.

**Figure 16:**
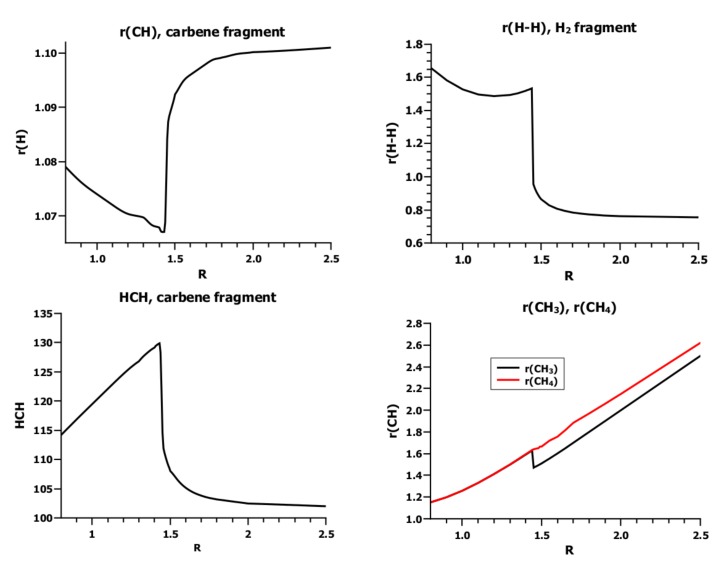
Carbene insertion into H_2_. Dependence of the geometry parameters on *R*. Energy in Hartrees; *R* in Å.

The geometry parameters of the fragments (Figure 16) show, however, that at this distance, an electron or spin rearrangement occurs with significant implications for the fragment geometries. At large *R* values, the HCH angle and C–H bond length are typical for carbene in the ^1^*A*_1_ state; 1.107 Å and 102.4 deg [26]. At *R* = 1.5 Å, both parameters change in a discontinuous way to values typical for carbene in the ^3^*B*_1_ state. At the same distance, the bond length of the hydrogen molecule doubles, which is impossible when the molecule is bonded. The distances between the carbon atom and the two hydrogen atoms are different in the initial phase of the insertion reaction, *i.e.*, when *R* ≥ 1.5 Å, the carbon is not pointing to the H_2_ midpoint; rather, the carbene and the hydrogen molecules approach each other in a parallel fashion, with the carbon atom closer to one hydrogen atom in H_2_ than to the other. At *R* = 1.5 Å the two C–H distances are equal, and the carbene has rotated from a parallel to a perpendicular position with respect to the hydrogen molecule, so the symmetry changes from *C*_*s*_ to *C*_2*v*_.

Many of the 20 FO-CSFs have weights less than 0.1 along the whole reaction coordinate; when all of these CSFs are neglected, eight CSFs remain, of which only three have significant weights. These CSFs are important at very different parts of the reaction coordinate. At large distances, the dominant CSF is NB, which describes the doubly-occupied s lone pair orbital on the carbon atom and the doubly-occupied σ MO in H_2_. At short distances, where the lowest excited carbene triplet state ^3^*B*_1_ is coupled with the lowest excited H_2_ triplet state to an overall singlet, TT dominates. Additionally, in between these two regions, around *R* = 1.5 Å, CSF X is important, where the hydrogen molecule is in its ground state, σ^2^, and the carbene is in the excited singlet state ^1^*B*_1_. This CSF has zero weight at large, as well as at small *R* values; it appears when bonding starts and disappears again when bonding is finished. Its role is to prepare the carbene in the ^1^*A*_1_ state for bonding. According to basic chemical principles, covalent bonding between fragments is only possible if unpaired electrons are available. Accordingly, the two singlet coupled electrons in the s lone pair orbital cannot contribute to covalent bonding, whereas the electrons in the triplet state can. However, this needs an excitation of one electron from the s to the p orbitals and a spin flip of one electron. The excitation without spin flip is described by X. Spin flip in one fragment needs spin flip in the second to guarantee that the ground-state multiplicity of the molecular wave function is not changed. As soon as this occurs, TT dominates, and X no longer describes a physical process in the system and disappears. The lowest triplet state of H_2_ is dissociative, which is in accord with the sudden increase in bond length when TT becomes dominant.

**Figure 17:**
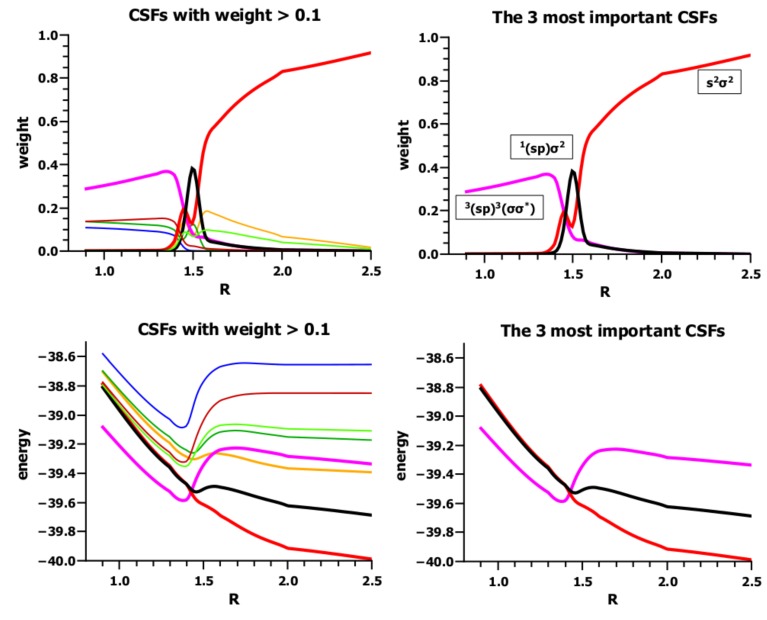
Carbene insertion into H_2_. **(Left)** Energies and weights of all FO-CSFs with weight larger than 0.1. **(Right)** Energy and weights of the three most important FO-CSFs. Energy in Hartrees; *R* in Å.

The shape of the energy curves (Figure 17) supports this interpretation: NB describes the two fragments each housing a pair of singlet-coupled active electrons. In singlet pairs, the two electrons have different spins, so they do not avoid each other as strongly as two electrons with like spins would. Accordingly, the electrons can come much closer to each other; the Coulomb repulsion increases; and the electron density is more extended than when electrons avoid each other due to spin correlation. To reduce the Coulomb repulsion of the electrons in the carbon 2s AO, one electron must be farther away from the carbon nucleus than the other (in-out correlation), which is possible when the 2s AO is more expanded; thus, contraction of the atomic orbital is unfavorable, and the energy of the NB CSF increases. The same can be said for the σ orbital in the H_2_ fragment. In X, only the hydrogen σ orbital is doubly occupied, but the two active electrons at the carbon atom occupy the orthogonal 2s and 2p_*π*_ AOs (angular correlation), which helps them to avoid each other, and therefore, a contraction of these two AOs is much less unfavorable than when the two electrons are in the same AO. For all other CSFs, contraction of the active FOs is favorable as a result of the two local spin flips.

## 4. What We Can Learn from the OVB Analysis

In all reactions where the reactants are in low-lying singlet states (low-spin states), the energies of all CSFs, but NB, decrease suddenly at a certain geometry. At the same geometry, the energy of NB increases, although this effect is less pronounced than the sudden energy decrease of the other CSFs. Concomitantly, the weights of the lowest high-spin states of the reactants coupled to a resultant low spin state increase strongly. If the system’s wave function is dominated by NB at large distances, the weight of this CSF decreases. This is not found for the carbene dimerization in *D*_2*h*_, where both reactants are already in their corresponding lowest high-spin state and where NB therefore has zero weight throughout the whole reaction. This finding is in accord with Lewis’ idea that covalent bonding between reactants is only possible if both fragments have unpaired electrons that can be singlet coupled to an electron pair. In the cases of carbene and silylene, the two electron spins must be coupled to a local high-spin state, which is only possible when they occupy the s and the p lone pair orbitals and are thus angularly correlated. Furthermore, the carbene insertion reaction shows that the high-spin state of the hydrogen molecule is best represented by a triplet excitation of the doubly-occupied σ MO, in which the two unpaired electrons are left-right correlated. The ^1^*A*_1_ state of the carbene reactant in this reaction is an excited fragment state, so the change from the singlet to triplet state is indeed a de-excitation, a process that seems to occur in two steps: First, the fragment goes from the low-lying ^1^*A*_1_ state to the higher lying ^1^*B*_1_ state; the second step is a spin flip in both fragments. The ^1^*B*_1_ state only helps to prepare the singlet carbene for bonding; at the equilibrium geometry, its weight is already zero. That such a CSF is important can only be seen when the whole reaction is investigated, not when an OVB analysis is only made at the equilibrium geometry.

It is noteworthy that, for all four reactions, the weight of neutral TT at the equilibrium distance is always smaller than 0.4, even when it is very large at intermediate distances, as in the case of the carbene dimerization. On the other hand, there are several ionic CSFs at the equilibrium geometry that, together, have a much larger weight than the neutral CSFs. The sum of the weights of the ionic CSFs in the set of the most important CSFs is at least as large as the weight of neutral TT.

When the reactions are described with the FORS wave function based on delocalized MOs, the fragment states in the four molecular systems are hidden, but some of the geometry parameters of the fragments may be helpful indicators for the fragment states. This point of view is not adopted by those who deny that, using such wave functions, one can make physical statements about fragment states in interacting systems, even when the fragments manifestly do not interact. According to this, only when isolated fragments are studied, one can say that the fragment geometry is caused by the fragment state; in all other cases, the origin of fragment geometries is not determined. For someone who denies that local states are responsible for fragment properties, local fragment states are theoretical entities; on the other hand, for someone who claims that the agreement of fragment geometries and fragment states, as suggested by the FO-CSFs, is too systematic to be just an accidental coincidence (as I do), speaking about fragment states and the information about local spin and charge distribution is speaking about entities that are as real as the free molecules that we are investigating. Monitoring geometry parameters during covalent bonding in a system suggests which local processes might be important for explaining what causes bonding. OVB reading of a proper FORS wave function should then describe these processes in more detail.

## 5. The Differences between Conventional VB and OVB

### 5.1. The Basis of Conventional VB

The Heitler–London calculation on H_2_ is based on the minimization of the expectation value of the molecular Hamiltonian:

$$\hat{H}={\hat{H}}_{A}\left(1\right)+{\hat{H}}_{B}\left(2\right)+V_{B}\left(1\right)+V_{A}\left(2\right)+\frac{1}{r_{12}}+\frac{1}{R}$$

with:

$${\hat{H}}_{A}\left(1\right)=\hat{T}\left(1\right)+V_{A}\left(1\right)\;V_{A}\left(1\right)=\frac{-1}{r_{1A}}\text{ analogoous for Atom B}$$

using a two-electron wave function (geminal) of the form:

(1) $$\Psi_{HL}\left(1,2\right)=\frac{1}{\sqrt{2\left(1+S^{2}\right)}}\left(a\left(1\right)b\left(2\right)+b\left(1\right)a\left(2\right)\right)$$

where *a* and *b* are the 1s AOs of the free hydrogen atom placed at hydrogen atoms A and B; the distance between atoms is *R*. The spatial part of the Heitler-London (HL) wave function Ψ_HL_ has Σg+1 symmetry.

The energy expectation value calculated with Ψ_HL_ is given by:

$$E_{HL}=\langle \Psi_{HL}\left|\hat{H}\right|\Psi_{HL}\rangle =2\langle {\hat{H}}_{A}\rangle +2\frac{J+KS}{1+S^{2}}+\frac{j+k}{1+S^{2}}+\frac{1}{R}$$

with:

$$E_{A}=\langle {\hat{H}}_{A}\rangle =\langle 1s_{A}\left|{\hat{H}}_{A}\right|1s_{A}\rangle ,$$

$$J=\langle 1s_{A}\left|V_{B}\right|1s_{A}\rangle ,$$

$$K=\langle 1s_{A}\left|V_{B}\right|1s_{B}\rangle ,$$

$$j=\langle 1s_{A}1s_{B}\left|\frac{1}{r_{12}}\right|1s_{A}1s_{B}\rangle ,$$

$$k=\langle 1s_{A}1s_{B}\left|\frac{1}{r_{12}}\right|1s_{B}1s_{A}\rangle$$

*J* is the Coulomb interaction between one electron and the other nucleus, the sum *j* + *k* is the Coulomb interaction between two electrons; *j* describes the Coulomb repulsion between the electrons located on the atoms; *k* is frequently described as the repulsion of two non-local charge distributions, also called exchange charge densities. Indeed, the sum of the two integrals describes the Coulomb repulsion between two independent charged Fermions. *KS*, on the other hand, is the contribution arising from the interference of the two hydrogen AOs.

There are three other wave functions that can be constructed with two hydrogen AOs:

$$\Psi_{I}\left(1,2\right)=\frac{1}{\sqrt{2\left(1+S^{2}\right)}}\left(a\left(1\right)a\left(2\right)+b\left(1\right)b\left(2\right)\right)$$

$$\Psi_{T}\left(1,2\right)=\frac{1}{\sqrt{2\left(1-S^{2}\right)}}\left(a\left(1\right)b\left(2\right)-b\left(1\right)a\left(2\right)\right)$$

$$\Psi_{S}\left(1,2\right)=\frac{1}{\sqrt{2\left(1-S^{2}\right)}}\left(a\left(1\right)a\left(2\right)-b\left(1\right)b\left(2\right)\right)$$

Ψ_I_ is the spatial part of the so-called ionic wave function of Σg+1 symmetry; Ψ_T_ is the spatial part of the Σu+3 wave action; and Ψ_S_ is the spatial part of the wave function describing the ionic Σu+1 state.

**Figure 18:**
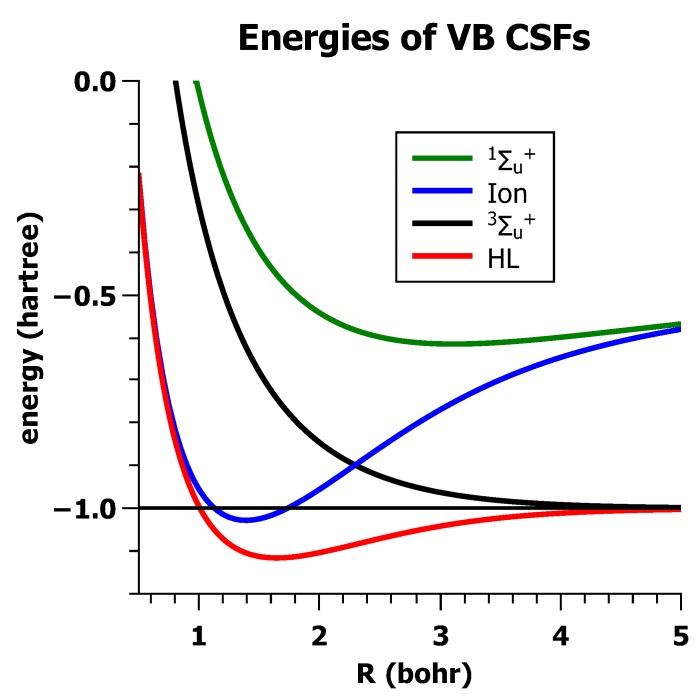
The energies of the four VB wave functions.

The energy expectation values for all four wave functions are shown in Figure 18; clearly, only the HL wave function Ψ_HL_ describes the stable H_2_ ground state qualitatively correctly. The energy curve of the ionic wave function does have a local minimum, but at a too-large equilibrium distance, and the stabilization energy with respect to two isolated hydrogen atoms is close to zero. The triplet-energy curve is completely repulsive, and the energy curve of the second ionic wave function lies very high. Nevertheless, the quantitative agreement between the experimental results and the theoretical results obtained with the HL wave function is poor.

One finds an equilibrium distance of *R*_*e*_ = 0.8679 Å and a dissociation energy of *D*_*e*_ = 304.5 kJ/mol; the best experimental values are *r*_*e*_ = 0.74117 Å and *D*_*e*_ = 456.8 kJ/mol, so the predicted equilibrium distance is 17% too long and the dissociation energy is 33% too small.

Since the HL and the ionic wave functions have the same Σg+1 symmetry, one can make linear combinations of them, *i.e.*, construct a CI (configuration interaction) wave function and do a CI calculation to get a better description of the Σg+1 ground state and also of the Σg+1 excited state (*vide infra*). However, this improves the description of the ground state only slightly; the ground state CI wave function Ψ = *c*_HL_Ψ_HL_ + *c*_I_Ψ_I_, the so-called Weinbaum function, is dominated by the HL wave function for all interatomic distances *R* from very large values to distances smaller than the equilibrium distance, as the absolute value of the CI coefficient of the HL wave functions is always much larger than that of the ionic wave function, |*c*_HL_| >> |*c*_I_|. Using the Weinbaum function, the equilibrium distance becomes even worse, *R*_*e*_ = 0.884 Å, while the dissociation energy improves slightly, *D*_*e*_ = 311.6 kJ/mol. These results are obtained with 1s AOs for the free hydrogen atom; when a basis function *χ*(*r*) = *Ne*^−*ζr*^ with a variational parameter *ζ* is used instead, the results are considerably improved: using a simple HL wave functions and *ζ*_opt_ = 1.17 gives *R*_*e*_ = 0.7356 Å and *D*_*e*_ = 364.7 kJ/mol; when *ζ* is optimized with the energy of the Weinbaum function, the equilibrium distance is *R*_*e*_ = 0.757 Å and *D*_*e*_ = 388.0 kJ/mol. By adding a p polarization function to the 1s AO with the optimized *ζ*, the results can again be improved: *R*_*e*_ = 0.746 Å and *D*_*e*_ = 397.5 kJ/mol with errors of 0.7% for the bond distance and 13% for the dissociation energy. These results seem to justify the view that it is the HL wave function that describes the major part of the stabilizing processes in the hydrogen molecule; all other contributions give just minor improvements.

This property of Ψ_HL_ is frequently related to the form of the wave function in which each AO is occupied by exactly one valence electron, which is thought to exactly represent covalency: Each atom contributing one electron to the bonding electron pair. Ψ_HL_ is therefore also called a covalent or a neutral wave function, and the same holds true for the triplet wave function Ψ_T_. The ionic wave functions, on the other hand, describe a cation/anion pair, they differ in their relative phases and in their symmetry. Ψ_HL_ and Ψ_I_ describe mutually exclusive electron distributions at large distances; the two wave functions are orthogonal to each other at large distances. At intermediate and short distances, the eigenfunctions of the (2,2)-CI problem are linear combinations of Ψ_HL_ and Ψ_I_.

Slater and Pauling generalized Heitler and London’s method to what is now called the (conventional) VB method, where the major role is played by covalent CSFs and where ionic CSFs are only used to correct some shortcomings of a treatment based solely on covalent CSFs. The interpretative power of VB methods has been convincingly shown by Shaik and Hiberty in several publications; see, for example, [27].

### 5.2. The Non-Orthogonality of VB-CSFs

**Figure 19:**
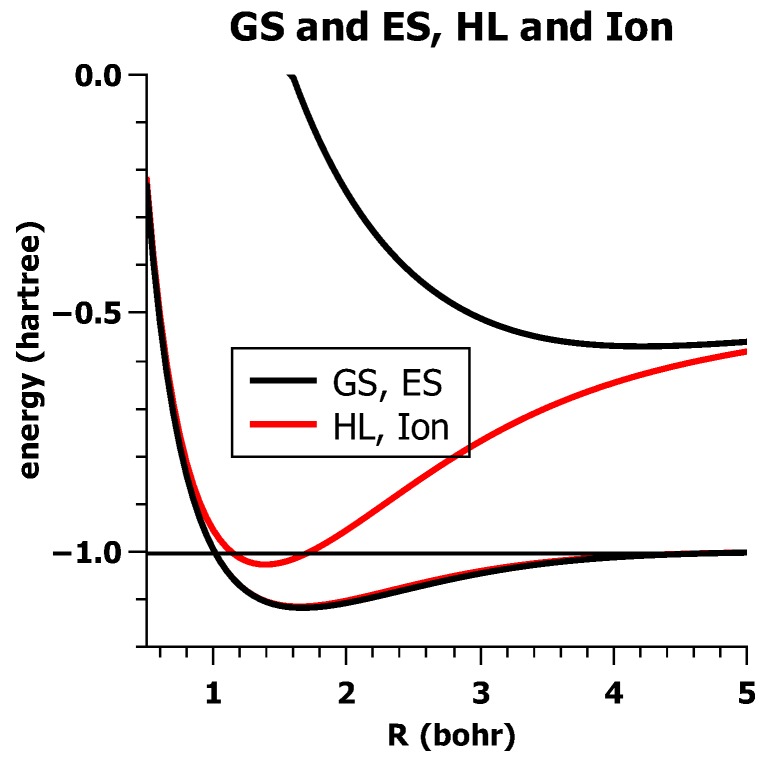
The energies of the ground state and the excited state and for the HL and the ionic CSF. Energy in Hartrees; bond length in Å.

In Figure 19, the CI-energies for the ground state *E*_*GS*_ and for the excited state *E*_*ES*_ are shown together with the energies of the two CSFs, *E*_HL_ and *E*_I_.

**Figure 20:**
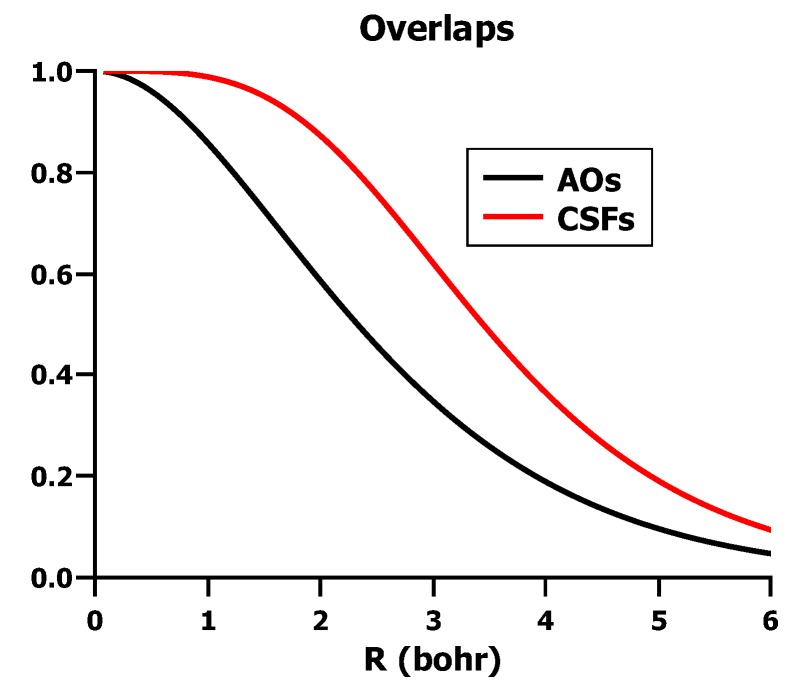
The overlap between the VB CSFs and between the hydrogen 1s AOs.

One can see that the ground state energy curve *E*_GS_ is nearly identical to the *E*_HL_ curve, whereas the energy curves *E*_ES_ and *E*_I_ only get close for large distances. This means, whereas Ψ_HL_ describes the ground state very well, the excited state is not dominated by Ψ_I_, except at large distances. One can also see that, for short distances, the energy curves of *E*_HL_ and *E*_I_ get very close and become identical for very small distances. This is due to the fact that Ψ_HL_ and Ψ_I_ are not orthogonal to each other; the overlap between the two wave functions is:

(2) $$\langle \Psi_{HL}|\Psi_{I}\rangle =\frac{2S}{1+S^{2}}$$

and this goes to one when *R* goes to zero, because the overlap of the hydrogen AOs *S* = 〈*a|b*〉 then goes to one. Figure 20 shows that the overlap between the VB wave functions approaches one much faster than the overlap between the AOs; at the equilibrium distance, their overlap is already greater than 0.95.

Minimization of the ground state energy using the Weinbaum function leads to an eigenvalue problem with (2,2)-matrices (a (2,2)-CI problem). Because of the non-orthogonality of the CSFs the CI problem is a generalized eigenvalue problem HC = SCE with the CI matrix H and the metric S.

$$H=\left(\begin{array}{cc} \langle \Psi_{HL}\left|\hat{H}\right|\Psi_{HL}\rangle & \langle \Psi_{HL}\left|\hat{H}\right|\Psi_{I}\rangle \\ \langle \Psi_{I}\left|\hat{H}\right|\Psi_{HL}\rangle & \langle \Psi_{I}\left|\hat{H}\right|\Psi_{I}\rangle \end{array}\right)$$

$$S=\left(\begin{array}{cc} 1 & \langle \Psi_{HL}|\Psi_{I}\rangle \\ \langle \Psi_{I}|\Psi_{HL}\rangle & 1 \end{array}\right)$$

$$C=\left(\begin{array}{c} c_{HL}\\ c_{I} \end{array}\right)$$

C is the matrix of the CI coefficients (eigenvectors), and E is the diagonal matrix of eigenvalues.

**Figure 21:**
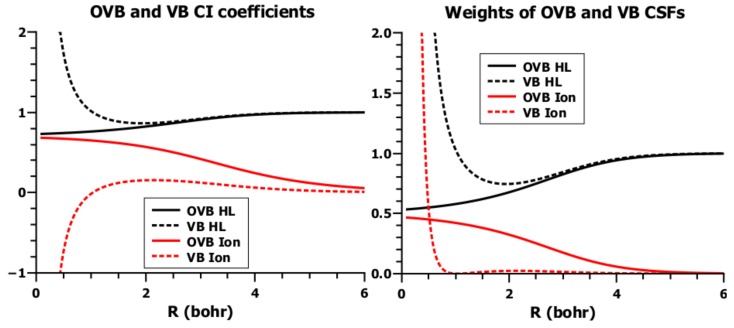
The CI coefficients and their squares of the (2,2)-CI problem using VB and OVB CSFs.

For *R* → 0, all four elements of the CI matrix become identical, and the outer diagonal matrix element of the metric becomes one, which means both matrices become singular. The CI coefficients and the squares for the two VB CSFs (Figure 21) show how differently ground and excited states are treated in conventional VB. For the ground state at distances larger than about *R* = 0.5 Å, the squares of the CI coefficients of Ψ_ion_ and Ψ_HL_ are nearly zero and nearly one, respectively, confirming that the ground state is well represented by the HL CSF alone. At distances smaller than the equilibrium distance when both matrices become singular, the CSFs are becoming linearly dependent and the CI coefficients of the CSFs are approaching infinity. Since the CI vectors of the generalized eigenvalue problem are orthogonal with respect to the metric S, the squares of the CI coefficients are not proper weights of the CSFs; instead, the Chirgwin–Coulson weights are mostly used to describe molecular electronic structures by their fractional ionic character [28]. However, since Ψ_HL_ describes two neutral hydrogen atoms only at large distances, but the same cation/anion pair as Ψ_I_ at small distances, such a characterization does not have unique physical relevance. Instead, the question arises: What does it mean calling Ψ_HL_ a covalent wave function, when it describes a neutral situation only at large distances, but an ionic one at small distances? One might as well claim that at small distances, Ψ_I_ is covalent. One immediate consequence is: From the form of a wave function, one cannot infer what kind of electron distribution it describes. This is in contrast with common belief: *In the valence bond (VB) view. . . , the electrons are viewed to interact so strongly that there is negligible probability of finding two electrons in the same orbital. The wave function is thus considered to be dominated by purely covalent contributions in which each electron is spin paired to another electron* [29]. Another consequence is: The non-orthogonality of VB CSFs poses difficulties for the interpretation of wave functions that are more severe than the numerical problems of the VB method frequently mentioned.

Nevertheless, even nowadays, chemists, who at best learned that VB is an obsolete method, often characterize the electron structure of molecules by the fractional ionic character of the state function, assuming or having heard that these numbers have physical relevance.

### 5.3. The Role of Interference in Conventional VB

**Figure 22:**
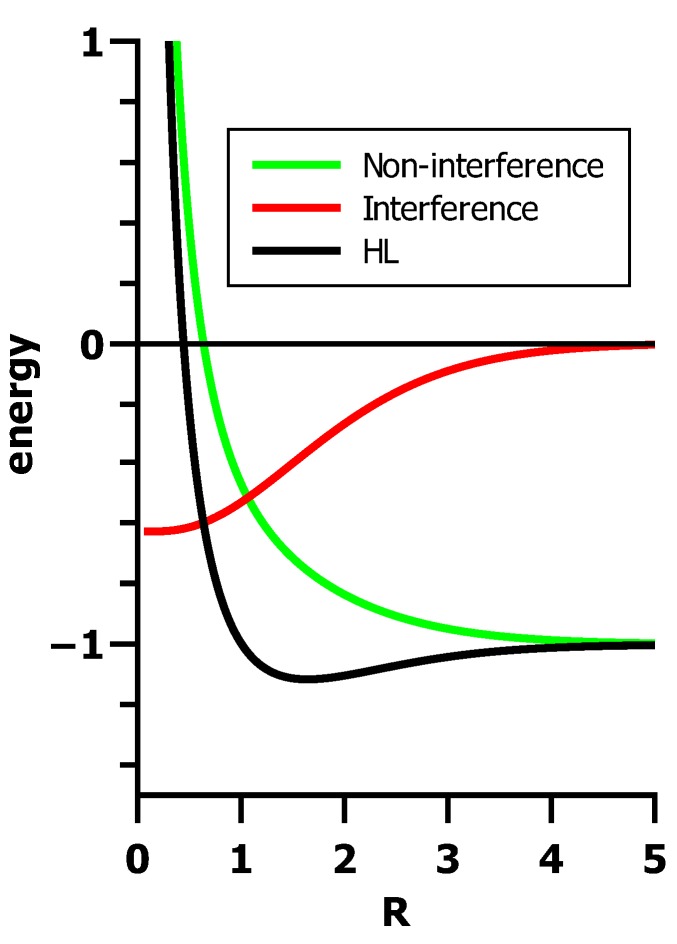
Partitioning of the Heitler–London energy into classical contributions and contributions caused by interference.

Why is Ψ_HL_ so well suited to describe bonding in H_2_? To answer this question, we reorder the contributions in the energy expression. The separation of the energy into classical and interference contributions shows (Figure 22) that only the interference of the non-orthogonal AOs causes bonding; the energy curve for the classical contributions without interference contributions is purely repulsive.

$$E_{HL}=\underset{\text{non-interference term}}{\underset{︸}{2\langle {\hat{H}}_{A}\rangle +2J+j+k+\frac{1}{R}}}+\underset{\mathrm{interference}\text{ term}}{\underset{︸}{\frac{-2\left(JS+K\right)S-\left(j+k\right)S^{2}}{1+S^{2}}}}$$

To be more precise: Constructive interference is responsible for bonding in the ground state of H_2_; the repulsive character of the triplet state is due to destructive interference. This can be seen from the one-particle densities normalized to the number of particles:

$$\rho_{HL}=\frac{1}{1+S^{2}}\left(1s_{A}^{2}+1s_{B}^{2}+2S1s_{A}1s_{B}\right)$$

$$\rho_{T}=\frac{1}{1-S^{2}}\left(1s_{A}^{2}+1s_{B}^{2}-2S1s_{A}1s_{B}\right)$$

When *R* approaches zero, then *S* approaches one, and therefore, the atomic contributions approach 0.5 in the HL CSF, whereas the interference contribution, which piles up charge in the internuclear region, approaches one. In the triplet wave function, the atomic density contributions are strongly positive, while the destructive interference contribution is strongly negative. As a consequence, the electron densities are located at the nuclei with their maxima outside the internuclear region. This is in accordance with the Pauli principle, which states that fermions with like spin avoid being spatially close. However, to call both wave functions covalent, although their electron and spin distributions are completely incompatible, shows that the word ‘covalent’ has conflicting semantics with respect to electronic wave functions.

The one-particle densities made with the other two VB CSFs do not add anything new; they are ρ_I_ = ρ_HL_ and ρ_S_ = ρ_T_. Therefore, both ${\text{Σ}}_{g}^{+}$ states show constructive interference, whereas both ${\text{Σ}}_{u}^{+}$ states show destructive interference.

This explanation of bonding in the hydrogen molecule has one flaw: It is based on the interference of atomic orbitals that never change their shape even though the atoms strongly interact. Chemical bonding is the result of strong interactions between atoms: The valence electrons of one atom are attracted by the nucleus of the other atom; the electrons repel each other due to charge and, also, if there are more than two electrons in the system, due to the Pauli principle. The electron density should reflect the results of these interactions: the electron density of one atom surrounded by other atoms must be more contracted due to the electron-electron repulsion, and the electron density must deviate from spherical symmetry due to polarization and repulsion. If a minimal basis with a single Slater function *χ*(*r*) = *Ne*^−*ζr*^ is used, contraction of the electron density can be accounted for by a variable exponent *ζ*, and polarization may be represented by a non-spherical AO by adding a p-type basis function to the 1s AO. Such an AO is similar to hybrid AOs. Calculations with such modified AOs were done in the early days of quantum chemistry, but they were always just seen as a way to improve the quantitative agreement between calculated and experimental data. To explain chemical bonding, the spherical 1s AOs of free hydrogen atoms were considered to be sufficient.

Coulson and Fischer [30] showed that it is possible to represent the ground-state wave function from the (2,2) CI problem by a single function of the HL-type, if the atom-centered AO *a* is replaced by a linear combination *ϕ*_*a*_ = *N* (*a* + *ϵb*) and *b* is replaced by the linear combination *ϕ*_*b*_ = *N* (*b* + *ϵa*) with a small positive *ϵ* depending on the interatomic distance and a normalization coefficient. These so-called semi-localized AOs are nodeless and non-orthogonal; they are the basis of the generalized VB method (GVB) by Goddard [31].

### 5.4. Orthogonal VB

Orthogonal AOs are frequently seen just as a convenient technical means in quantum chemical calculations. Symmetric orthonormalization of AOs was introduced into solid-state physics by Wannier [32] and into molecular quantum theory by Löwdin [33], and even when the AOs used in quantum chemical calculations were not orthogonal, they were assumed to be, as in the Pariser–Parr–Pople (PPP) method [34,35,36], or quite general in the zero differential overlap (ZDO) approximation [37,38,39]). No wonder that symmetrically orthonormalized AOs (OAOs) were also used in VB calculations on H_2_ [40,41].

$$a=\frac{1}{2}\left(\frac{1}{\sqrt{1+S}}+\frac{1}{\sqrt{1-S}}\right)1s_{A}+\frac{1}{2}\left(\frac{1}{\sqrt{1+S}}-\frac{1}{\sqrt{1-S}}\right)1s_{B}=P1s_{A}+Q1s_{B}$$

$$b=\frac{1}{2}\left(\frac{1}{\sqrt{1+S}}-\frac{1}{\sqrt{1-S}}\right)1s_{A}+\frac{1}{2}\left(\frac{1}{\sqrt{1+S}}+\frac{1}{\sqrt{1-S}}\right)1s_{B}=Q1s_{A}+P1s_{B}$$

An unexpected result of these calculations was that the energy curve for the HL-type wave function ${\text{Ψ}}_{\mathrm{HL}}^{o}$ (where the superscript *o* indicates a wave function made with OAOs) is completely repulsive, like that for the triplet state; indeed, the two energy curves are parallel, with the HL-curve lying slightly above the triplet curve. Similarly, the energy curve of the ionic wave function of ${\text{Σ}}_{g}^{+}$ symmetry is parallel to the energy curve of the ionic Σu+1; again, the former lying slightly above the latter. For the two ${\text{Σ}}_{u}^{+}$ states, one finds that the wave functions and, accordingly, the energy curves are completely unchanged when the non-orthogonal AOs are replaced by OAOs.

Since the OAOs depend linearly on the AOs, so do the CSFs. For the ${\text{Σ}}_{g}^{+}$ CSFs, the linear transformations are [42]:

$$\Psi_{HL}^{o}=\sqrt{\frac{1+S^{2}}{1-S^{2}}}\left(\Psi_{HL}^{n}-S\Psi_{I}^{n}\right)$$

$$\Psi_{I}^{o}=\sqrt{\frac{1+S^{2}}{1-S^{2}}}\left(\Psi_{I}^{n}-S\Psi_{HL}^{n}\right)$$

$$\Psi_{HL}^{n}=\frac{1}{\sqrt{1+S^{2}}}\left(\Psi_{HL}^{o}+S\Psi_{I}^{o}\right)$$

$$\Psi_{I}^{n}=\frac{1}{\sqrt{1+S^{2}}}\left(\Psi_{I}^{o}+S\Psi_{HL}^{o}\right)$$

where VB-CSFs are labeled with the superscript *n*; for the ${\text{Σ}}_{u}^{+}$ CSFs, it holds:

$$\Psi_{T}^{o}=\Psi_{T}^{n}$$

$$\Psi_{S}^{o}=\Psi_{S}^{n}$$

Because ${\text{Ψ}}_{\mathrm{HL}}^{n}$ and ${\text{Ψ}}_{\text{I}}^{n}$ are a non-orthogonal basis for the (2,2)-CI problem, ${\text{Ψ}}_{\mathrm{HL}}^{o}$ and ${\text{Ψ}}_{\text{I}}^{o}$ are an orthogonal basis for the same CI problem; the results for ground and excited states are the same, irrespective of which basis is chosen. Only when individual basis vectors are compared do the differences between VB and OVB become apparent. The difference between ${\text{Ψ}}_{\mathrm{HL}}^{o}$ and ${\text{Ψ}}_{\mathrm{HL}}^{n}$ is best seen by comparing their one-particle densities. It turns out that $\rho_{\mathrm{HL}}^{o}$ = $\rho_{\text{T}}^{n}$, which means ${\text{Ψ}}_{\mathrm{HL}}^{o}$ shows the same destructive interference as does ${\text{Ψ}}_{\text{T}}^{o}$. Additionally, this is due to the use of OAOs, as has been long known. To quote Slater: *This* *is not surprising; for our discussion of the nature of the covalent bond . . . has made it clear that it is the overlap charge which is responsible for the binding, and these orthogonalized orbitals are just set up so as to avoid overlap. If one uses them, one can still carry out a configuration interaction and end up with the same results which we have obtained by our other methods* [43].

Slater pointed to the fact that orthogonalization of AOs prevents constructive interference in ${\text{Ψ}}_{\mathrm{HL}}^{o}$, and we showed that all OVB-CSFs have the same one-particle density. Proper linear combinations of ${\text{Ψ}}_{\mathrm{HL}}^{o}$ and ${\text{Ψ}}_{\text{I}}^{o}$ describe the bonded ground state of the H_2_ molecule as correctly as do linear combinations of ${\text{Ψ}}_{\mathrm{HL}}^{n}$ and ${\text{Ψ}}_{\text{I}}^{n}$, regardless of the fact that the one-particle density of the former CSFs shows destructive interference and that of the latter shows constructive interference. The correct description of bonding does not depend on a certain choice of AOs.

**Figure 23:**
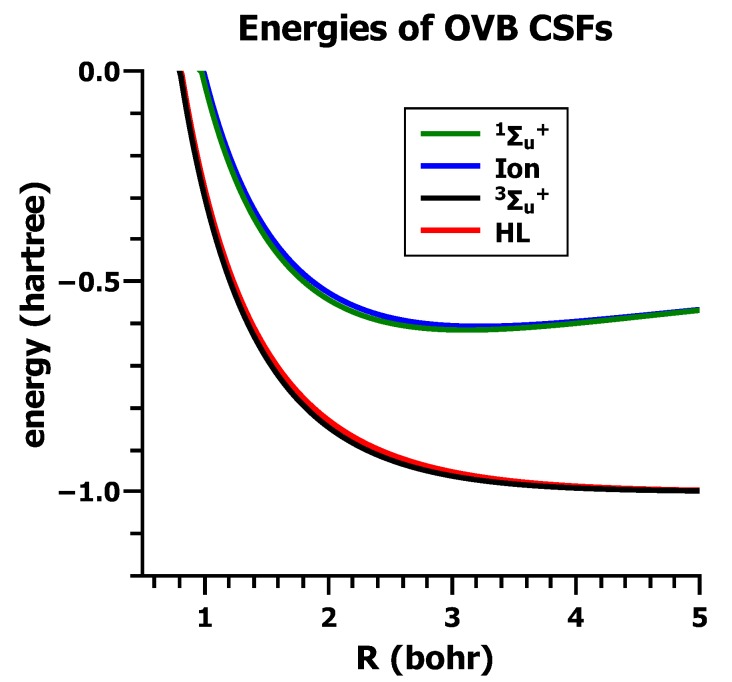
The energy curves for the orthogonal configurational state functions.

A second fact is also well known from McWeeny’s work in the 1950s [41]: The VB-CSF ${\text{Ψ}}_{\mathrm{HL}}^{n}$ is a linear combination of the neutral CSF ${\text{Ψ}}_{\mathrm{HL}}^{o}$ and the ionic CSF ${\text{Ψ}}_{\text{I}}^{o}$, and since ${\text{Ψ}}_{\mathrm{HL}}^{n}$ is a very good description of the H_2_ ground state, the linear combination of OVB CSFs is also a very good description of the ground state. Because the OVB CSFs are orthogonal to each other, the neutral CSF can never describe an ionic electron distribution and *vice versa*. However, since the overlap integral *S* increases with decreasing distance *R*, the ionic contribution increases, as well. At large distances *R*, where *S* = 0, ${\text{Ψ}}_{\mathrm{HL}}^{n}$ is identical to ${\text{Ψ}}_{\mathrm{HL}}^{o}$, and the electron distribution in the ground state is strictly neutral or covalent. With increasing *S*, the ionic contribution increases, as well, but the contribution of ${\text{Ψ}}_{\mathrm{HL}}^{o}$ is always larger than that of ${\text{Ψ}}_{\mathrm{ion}}^{o}$. For *R* approaching zero, both coefficients of the linear combination approach 1/$\sqrt{2}$, which means the weight of covalent and ionic CSF is 1/2 for *R* = 0 (Figure 23). Pilar [44] in his *Elementary Quantum Chemistry* summarized the findings by McWeeny as follows: *This means that the concepts of covalent and ionic character are not unique. In using the Slater–Pauling method for polyatomic molecules, it has been standard practice among chemists to speak of the relative importance of ionic structures in a molecule in terms of the coefficients of the corresponding wave function in the total wave function. The above analysis shows that such an interpretation does not have a unique physical significance. Of course, this is not at all surprising in the light of a previous discussion. . . , where it was shown that the so-called covalent and ionic functions used to describe H*_2_ *have an overlap of* 0.95 *and thus have no unique interpretation in terms of fractional ionic character. One must then conclude that any Slater–Pauling covalent wave function which predicts stable chemical bonding does so only because the wave function contains ionic wave functions in terms of OAO’s. In conclusion, the use of OAO’s in the VB method leads to a clearer electrostatic picture of chemical bonding but destroys the chemist’s simple concepts of covalent and ionic character.* In light of the relation of VB and OVB CSFs and the implications for their interpretation (known for half a century), statements of the following kind are surprising: *. . . we may say that the symmetric orthonormalization gives very close to the poorest possible linear combination for determining the lowest energy. This results from the added kinetic energy of the orbitals produced by a node that is not needed.. . . We have here a good example of how unnatural orthogonality between orbitals on different centers can have serious consequences for obtaining good energies and wave functions* [45].

### 5.5. OVB and Chemical Bonding

The most profound analyses of chemical bonding are due to Klaus Ruedenberg. Starting in the 1960s, he and his coworkers showed, in a series of papers [46,47,48,49,50,51,52,53,54,55,56,57] , what can be summarized as Ruedenberg’s physical interpretation of covalent bonding in ${\text{H}}_{2}^{+}$:

(1) Covalent bonding is the result of the lowering of kinetic energy through inter-atomic electron delocalization, called electron-sharing. Delocalization is caused by constructive interference during the superposition of hydrogen AOs. The electrostatic interactions due to charge accumulation in the internuclear region are not bonding, as is frequently claimed, but debonding.
(2) Electron-sharing is accompanied by intra-atomic contraction and polarization. Contraction causes a decrease in the intra-atomic electrostatic energy and an increase in the intra-atomic kinetic energy in the deformed atoms in the molecule.
(3) Intra-atomic contraction enhances the inter-atomic lowering of the kinetic energy and, thus, contributes to energy minimization.
(4) The antagonistic changes of intra-atomic and inter-atomic energy contributions cause a variational competition between electrostatic and kinetic energy; the wave function that achieves the optimal total energy is obtained by variational optimization.
(5) The atom-centered orbitals describing the deformed atoms are quasi-AOs; their shape depending on the distance between the interacting atoms. Near equilibrium distance, they are more contracted than the free AOs, causing the lowering of electrostatic energy; at larger distances, they may be even more expanded than in the free atom, because then the electron can better expand into spatial regions not available for the electron in the free atom when the AOs are superimposed.

It was shown for the ${\text{H}}_{2}^{+}$ ion that the quasi-AOs are very similar to free AOs; the difference between them is not larger than 6% (measured by their overlap); the major deformation is contraction. One might assume that this deformation of the AOs during bonding is negligible; however, neglecting the deformation decreases the bonding energy by about 50% [53]. Moreover, of the 6% deformation, about 75% is contraction and only 25% is polarization. Nonetheless, the latter again has an unexpected impact: The Coulomb interaction between the proton (Atom A) and the spherically-contracted quasi-atomic density at Atom B is repulsive at all distances, but if the quasi-atomic density is also polarized, the Coulomb interaction is attractive at all distances.

It has been shown that the bonding in H_2_ is completely analogous; because of the additional electron-electron repulsion, the total bonding energy is not twice the bonding energy of ${\text{H}}_{2}^{+}$, but only 85% of it [53]. For the many-electron molecules B_2_, C_2_, N_2_, O_2_ and F_2_, the basic conclusions remain valid: Because of the larger number of interacting electrons, the deformation of the atoms in the molecule (due to electrostatic interactions or due to the Pauli exclusion principle) becomes more important, and the wave function adjustment may become a subtle problem.

**Figure 24:**
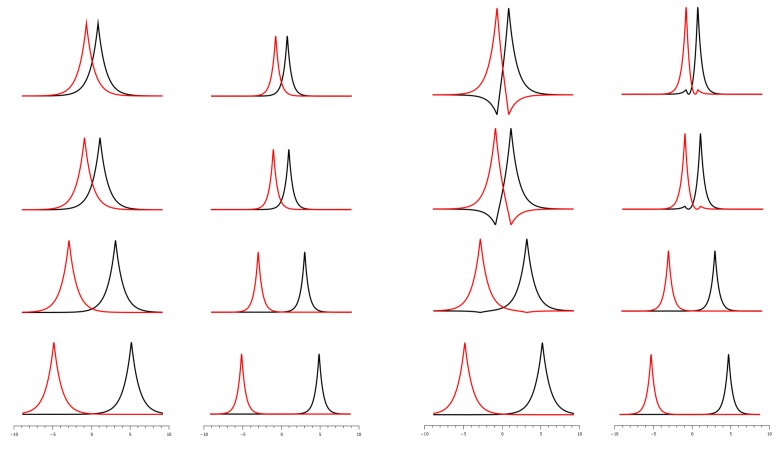
(**Left**) Shape of the 1s AOs and squared AOs for different atom distances; (**right**) shape of the 1s orthonormalized AOs (OAOs) and squared OAOs for different atom distances.

Ruedenberg’s analysis is based on non-orthogonal quasi-AOs allowing interference; in OVB, contraction and polarization of OAOs is enforced by orthogonalization, irrespective of whether 1s AOs are symmetrically orthogonalized or orthogonal MOs are localized on fragments by an orthogonal transformation. On the right side of Figure 24, one can see that symmetrical orthogonalization transforms the spherical 1s AOs into contracted sp-like hybrid orbitals. On the left side, one can see what is described by the original Heitler–London treatment: although the 1s AOs strongly interact with each other during bonding, they are not perturbed; instead, there is what is called mutual interpenetration of the never-changing electron densities. Accordingly, there is only interference of the 1s states of free hydrogen atoms, even at the equilibrium state of the hydrogen molecule. Obviously, this reaction is a model reaction of two fictitious hydrogen atoms; it demonstrates that interference is responsible for delocalization of the electrons and, thus, for electron sharing. Unless the AOs are scaled, or extremely large basis sets are used that can mimic the scaling, and polarization is allowed, e.g., by adding p-type AOs, then the influence of contraction and polarization on bonding is not accounted for. In OVB, on the other hand, the OAOs are made, as Slater has pointed out, to prevent any interference and, thus, electron sharing. Therefore, the neutral HL-type CSF cannot describe covalent bonding, regardless of whether the OAOs are contracted and polarized or not. Electron delocalization is only represented by the ionic CSF, and the ground-state wave function must be a linear combination of the neutral and ionic CSFs; at large distances, it is purely neutral, but with decreasing *R*, both CSFs become equally important (see Figure 21). In the Coulson–Fisher treatment, electron sharing is represented by increasingly delocalized non-orthogonal atom-centered AOs, while in OVB, delocalization is represented by the increasing weight of the ionic CSF. This is completely analogous to the Hubbard model [58,59] in solid-state physics, where each site contributes one electron occupying an orthogonal AO. The ground state is represented by a linear combination of VB-like CSFs; neutral CSFs describe electron distributions, where each site hosts a single electron; in ionic CSFs, some sites are occupied by two and other sites by zero electrons. In the OVB description of the H_2_ molecule, the outer diagonal element of the CI matrix, the coupling matrix element, describes the transfer of an electron from one atom to the other, thus changing the neutral electron distribution into an ionic one. The same role is played by the “hopping integral” in the Hubbard model (the author is grateful to Prof. Ruedenberg for having pointed out this fact).

### 5.6. Diabaticity of OVB CSFs

It is a characteristic of VB CSFs that they can change their character with their molecular geometry. In contrast to such chameleon-like state functions, the electron configurations described by OVB CSFs never change along the interatomic distance *R*: Neutral CSFs always describe neutral configurations, and ionic CSFs always describe ionic ones. This is reminiscent of the characterization of wave functions as adiabatic and diabatic. Adiabatic states are eigenstates of the molecular Hamiltonian, and wave functions representing adiabatic states are adiabatic wave functions. Whenever two or more potential energy surfaces closely approach each other in certain regions of configurational space, their energies and corresponding state functions undergo rapid changes in these regions, which causes problems not only in the determination of the electronic wave functions, but also in the treatment of reaction dynamics. In both cases, the resolution of adiabatic wave functions in terms of diabatic wave functions is a possible way out, because diabatic wave functions are much easier to handle and they do not rapidly change in the same spatial regions as adiabatic wave functions do. The drawback is that diabatic states are not eigenfunctions of the Hamiltonian, but must be constructed on the basis of criteria coming from the application field under consideration. There are two conceptually totally different approaches to constructing diabatic wave functions, as described by Atchity and Ruedenberg [60]. One is the dynamic approach, where *one has* *to deal with a set of coupled differential equations between the adiabatic states* with large coupling terms *(nuclear-derivative matrix* *elements between electronic states), and the construction of diabatic states is guided by the goal of* minimizing the coupling terms in the dynamic equations. Angeli *et al*. [61] calculated the coupling matrix elements between the neutral and ionic CSFs, both based on AOs and OAOs, and showed that the coupling matrix element was only zero for the OVB CSFs, but not for the VB CSFs.

In the second approach, the electronic structure approach, one starts with the observation that, in certain regions of coordinate space, *drastic changes occur in the electronic structures* of the adiabatic states, and the construction of diabatic states is guided by the goal of finding wave functions whose *electronic structures maintain their essential characteristics over the entirety of such regions*. In the following, the electronic structure approach, based on the maximization of configurational uniformity, as developed by Atchity and Ruedenberg, is presented [60]. This approach *requires the ability: (i) To quantitatively assess the character of the structure of a wavefunction in electronic coordinate space; and (ii) To monitor changes in these characteristics as functions of the nuclear positions over regions in nuclear coordinate space. When these characteristics change only little for a wavefunction in such a region, then we consider the electronic structure of that wavefunction uniform in that region.* This approach was designed for the investigation of a few states, say *N*, by means of state-averaged MCSCF wave functions, where it is assumed that the number of CSFs *M* is much larger than the number of states. If the MOs used to span the CSFs are unambiguously defined, the electronic structure of each state function can be characterized by the CI coefficients. Furthermore, it is assumed that each state function is dominated by only a few CSFs, and their CI coefficients characterize the electronic structure. It is furthermore assumed that, when a molecule deforms during a reaction, all involved MOs deform continuously along the reaction path in nuclear coordinate space, and since it is known that MOs only change their shape gradually, any rapid change in the electronic structure comes from strong changes in the CI coefficients as functions of the nuclear coordinates. Given this, *a wavefunction is considered to essentially maintain its electronic structure along a nuclear coordinate path if the deforming configurations in its dominant part remain the same. If this holds true for all points in a nuclear coordinate region, then we consider the state to have a uniform electronic structure in this region. In this sense, we equate electronic structure uniformity with configurational uniformity*. In regions where strong change in the electronic structure is observed, the adiabatic states will not exhibit configurational uniformity over the entire region. It is now assumed that the *M* CSFs can be partitioned into *N* configuration groups so that in any adiabatic wave function, all members of a certain configuration group are always either dominant or not dominant. If all of these conditions are met, one can surmise that the *N* adiabatic states can be expressed as linear combinations of *N* diabatic states, *each of which is dominated, everywhere in nuclear coordinate space, by the configurations of one and the same configuration group. . .*. The MOs that allow the construction of such diabatic states are called diabatic MOs (DMO). Nakamura and Truhlar [62,63,64] developed a method to determine DMOs, called the four-fold way, and investigated the deformation of several molecules during chemical reactions. They showed that good candidates for DMOs are orbitals localized on a few atoms.

The results for the H_2_ molecule by Angeli *et al*. [61] are in full agreement with the electronic structure approach: When the DMOs are OAOs, the adiabatic states are linear combinations of the neutral and ionic OVB CSFs, which show electronic uniformity along the whole reaction coordinate.

For the collinear reaction of two H_2_ molecules, Nakamura and Truhlar showed that the second and third excited singlet states can be characterized by the σ → σ^∗^ excitation in either of the two H_2_ molecules. Therefore, the DMOs must be FOs localized on each molecule. When the delocalized MOs, using the input data for this example, are Procrustes localized on the hydrogen molecules and these localized FOs are used as DMOs, the diabatic states are identical with those constructed with Nakamura’s DMOs. This show, that, at least for this example, the FOs are indeed DMOs. More investigations are necessary to find out whether or not OVB CSFs are always diabatic; investigations using the coupling matrix method will be made in Angeli’s group, and those using the electronic structure approach will be made in the group of Sax.

As mentioned, there are many possibilities to construct states with a varying degree of diabaticity, and often the diabatic character was claimed, but not proven. This is especially true for conventional VB CSFs, where neutral and ionic CSFs were often claimed to be diabatic (see, for example, [27]). In the group of Malrieu, a method was developed to construct from adiabatic CI wave functions nearly diabatic states that are as close as possible to conventional VB CSFs [65,66], and it was said that for intermediate nuclear configurations, the transformed wave functions resemble as much as possible the VB CSFs; one may therefore consider them as nearly diabatic [65]. Angeli *et al*. [61] showed that conventional VB CSFs are not diabatic; therefore, one can question the diabaticity of the transformed wave functions for intermediate nuclear configurations, unless it is proven explicitly.

## 6. Discussion

Local information about chemical reactions is, in general, hidden by the delocalized MOs that are used to construct high-quality wave functions. VB methods are made to reveal local information. Conventional Slater–Pauling VB wave functions based on non-orthogonal AOs hide the real electronic structure behind a seemingly neutral HL-type wave function; OVB wave functions also reveal this information. OVB reading of MCSCF wave functions is therefore an excellent means to get information about charge and spin redistribution during chemical reactions, especially reactions connected to chemical bonding. The OVB view of chemical bonding is different from the VB view, unless one is only interested in calculating energy curves [67].

The sample reactions discussed above demonstrate how the fragment properties determine the local processes in elementary reactions. During the dimerization of triplet carbene, for example, no local excitations are necessary to make the carbenes ready for bonding; each fragment is already in a local high-spin state, and therefore, TT CSF is the most important neutral CSF. Electron sharing during bonding is reflected by the increasing weights of the two ionics, CX1 and CX2; these two CSFs are necessary to describe the angular correlation of the electrons in the anion/cation pair. In the ethene molecule, angular correlation in the neutral electron distribution becomes possible by CSF DX.

The local processes during planar dimerization of singlet silylenes, on the other hand, are very different. At large distances, the two fragments are described by the no-bond configuration, while the angular correlation of the singlets is described by CSF DX. To make the fragments ready for bonding, they must be locally excited into the lowest triplet states, where the Fermi correlation of the two valence electrons causes a pronounced contraction of the electron distribution and a strong energetic stabilization, as can be seen from the kinks in all (but one) energy curves at 3.5 Å. Electron sharing is again described by the two ionic CSFs, CX1 and CX2. This reaction shows not only that preparing fragments for bonding often requires spin reorganization, it also shows that excitation into local high-spin states yields strong energetic stabilization. Additionally, one can see that spin reorganization processes are accompanied by drastic changes in the fragment geometries, like bond lengths or bond angles. The change in the HSiH angle from 92 to 118 degrees will certainly be seen by some chemists as an indication that sp^2^ hybrid orbitals are needed to correctly describe the equilibrium geometry of planar disilene, but this *post festum* argument completely ignores the information that we have for the spin reorganization in the reacting fragments during the dimerization reaction.

Comparison of planar and non-planar silylene dimerization shows how local processes also depend on the local symmetry. With no σ − *π* separation, preparation for bonding is also possible by a single-charge transfer, represented by CSF C, not just by excitation into a local high-spin state. For planar dimerization, CSF C does not have the symmetry of the ground state and can, therefore, not contribute, but as soon as the symmetry has been lowered, single-charge transfer becomes an important process during bonding. At very short distances, where the disilene molecule is again planar, the weight of CSF C is zero again. One can also see that, at much larger distances, the ionic CSF C becomes more important than the neutral CSF TT. Electron sharing is represented only by the ionic CSF CX1, which, at short distance, has the same weight as the most important neutral CSF TT. The local symmetry at the silicon atoms during bonding resembles that of a silyl radical; *i.e.*, the HSiH angles are closer to tetrahedral angles, which, again, some chemists prefer to explain with the help of sp^3^ hybrids.

For the insertion of singlet carbene into the hydrogen molecule, both reactants are in electronic states that are not prepared for bonding; *i.e.*, the carbene is in an excited state that corresponds to the silylene ground state. One could assume that, with simple de-excitation from the local ^1^*A*_1_ state into the local ^3^*B*_1_ state, the carbene is prepared for bonding, but as one can see, first, there is a local excitation into the ^1^*B*_*a*_ state, by which the singlet-coupled valence electrons residing in the s-type lone pair AO become distributed among the s- and p-AOs and (only) then does the spin flip occurs simultaneously in both the carbene and hydrogen fragments. In the carbene fragment, this process is a de-excitation into the triplet ground state, whereas in the hydrogen molecule, it is an excitation into the unbound triplet state. The Fermi correlation in the carbene triplet state again causes a strong contraction of the electron densities in the fragments and a strong energetic stabilization. Due to the low overall symmetry, more than two ionic CSFs describe the electron sharing and, thus, the major contribution to chemical bonding.

The traditional explanation of bonding based on hybrid orbitals has several drawbacks. First of all, argumentations based on hybrid orbitals are mostly used to explain the local symmetry at heavy atoms in the reaction product; only seldom are they used to explain the continuous geometry changes during the bonding process. Hybridization is the superposition of atomic eigenfunctions of different angular momenta under the influence of the potentials of all other atoms. The local symmetry of this perturbing potential determines the kind of hybridization; hybridization is thus the result of the molecular nuclear framework, not its cause. Therefore, if one wants to find the origin of the molecular geometry, one has to find the processes that cause the change in molecular geometries from the reactants to the product. Lennard–Jones [68] showed in the early 1950s that linear, trigonal and tetrahedral structures, as in ethyne, ethene and ethane, are compatible with the maxima of the probability of finding two, three or four particles with the same spins on a sphere. According to the Pauli exclusion principle, the spins avoid each other optimally, and the so-called Pauli repulsion is more important for the shape of molecules than electrostatic forces. In a realistic molecule, the spin distribution is not as isotropic as it is on a sphere, and therefore, the maxima of the spin distributions must be calculated with appropriate methods; e.g., quantum Monte Carlo (QMC) methods. Such calculations were made by Scemama *et al*. [69] and Lüchow [70]; the combination of OVB and QMC methods seems to be very promising for investigating the processes of chemical bonding. With such combined investigations, it should be possible to clarify the reality (or otherwise) of fragment states in a molecule.

## 7. Computational Methods

The CAS-SCF wave function for the model reactions was calculated with GAMESS [71] using the 6-31G* basis. The programs for creating the FOs were implemented in a local version of GAMESS. The H2 calculations using the symmetrically orthonormalized 1s AOs were performed by using a MATLAB program. The formulae were taken from Slater’s book [43].

## 8. Conclusions

When VB CSFs are made with orthogonal fragment orbitals, the CSFs themselves will also be orthogonal; therefore such CSFs describe electronic structures that maintain their characteristics over large regions of coordinate space. In other words, neutral CSFs remain neutral and ionic CSFs remain ionic; in contrast to non-orthogonal CSFs used in conventional VB. In this way, OVB describes all local processes occurring during chemical reactions much better than conventional VB. If orthogonal transformations are used to localize delocalized CASSCF MOs on predefined fragments, then CASSCF wave functions will be transformed into OVB wave functions, thereby revealing information that is otherwise hidden in wave functions made with both delocalized orthogonal and localized non-orthogonal orbitals. This is what OVB reading of a CASSCF wave function means.

## References

[1] Cartwright N. (1983). How the Laws of Physics Lie.

[2] Hacking I. (1983). Representing and Intervening.

[3] Giere R.N. (1999). Science without Laws.

[4] Giere R.N. (1988). Explaining Science. A Cognitive Approach.

[5] Gelfert A. (2003). Manipulative success and the unreal. Int. Stud. Phil. Sci..

[6] Frenking G. (2007). Unicorns in the world of chemical bonding models. J. Comp. Chem..

[7] Falkenburg B. (2010). Particle Methaphysics.

[8] Jeffrey G.A. (1997). An Introduction to Hydrogen Bonding.

[9] Stone A. (2013). The Theory of Intermolecular Forces.

[10] Hoja J., Sax A.F., Szalewicz K. (2014). Is electrostatics sufficient to describe hydrogen-bonding interactions?. Chem. Eur. J..

[11] Levine R.D. (2005). Molecular Reaction Dynamics.

[12] Schmidt M.W., Gordon M.S. (1998). The construction and interpretation of MCSCF wavefunctions. Ann. Rev. Phys. Chem..

[13] Roos B.O. (1987). The complete active space self-consistent field method and its applications in electronic structure calculations. Adv. Chem. Phys..

[14] Roos B.O., Roos B. (1992). The multiconfigurational (MC) self-consistent field (SCF) theory. Lecture Notes in Quantum Chemistry.

[15] Ruedenberg K., Sundberg K.R., Calais J.L., Goscinski O., Linderberg J., Öhrn Y. (1976). MCSCF studies of chemical reactions: Natural reaction orbitals. Quantum Science.

[16] Cheung L.M., Sundberg K.R., Ruedenberg K. (1978). Dimerization of carbene to ethylene. J. Am. Chem. Soc..

[17] Cheung L.M., Sundberg K.R., Ruedenberg K. (1979). Electronic rearrangements during chemical reactions II. Planar dissociation of ethylene. Int. J. Quantum Chem..

[18] Ruedenberg K., Schmidt Michael, W., Gilbert Mary, M., Elbert S.T. (1982). Are atoms intrinsic to molecular electronic wave functions? I . The FORS model. Chem. Phys..

[19] Ruedenberg K., Schmidt M.W., Gilbert M.M., Elbert S.T. (1982). Are atoms intrinsic to molecular electronic wave functions? III . Analysis of FORS configurations. Chem. Phys..

[20] Ruedenberg K., Schmidt M.W., Gilbert M.M. (1982). Are atoms intrinsic to molecular electronic wave functions? II . Analysis of FORS orbitals. Chem. Phys..

[21] Feller D.F., Schmidt M.W., Ruedenberg K. (1982). Concerted dihydrogen exchange between ethane and ethylene. SCF and FORS calculations of the barrier. J. Am. Chem. Soc..

[22] Sax A.F. (2012). Localization of molecular orbitals on fragments. J. Comp. Chem..

[23] Bunker P.R., Jensen P., Kraemer W.P., Beardsworth R. (1986). The potential surface of X ^3^*B*_1_ methylene (CH2) and the singlet-triplet splitting. J. Chem. Phys..

[24] Dubois I. (1968). The absorption spectrum of the free SiH−2 radical. Can. J. Phys..

[25] Balasubramanian K., McLean A.D. (1986). The singlet-triple energy separation in silylene. J. Chem. Phys..

[26] Petek H., Nesbitt D.J., Darwin D.C., Ogilby P.R., Moore C.B., Ramsay D.A. (1989). Analysis of CH_2_
*ã*^1^*A*_1_ (1,0,0) and (0,0,1) Coriolis-coupled states, *ã*^1^*A*_1_ − $\tilde{X}$^3^*B*_1_ spin-orbit coupling, and the equilibrium structure of CH_2_*ã*^1^*A*_1_ state. J. Chem. Phys..

[27] Shaik S.S., Hiberty P.C. (2008). A Chemist’s Guide to Valence Bond Theory.

[28] Chirgwin B.H., Coulson C.A. (1950). The electronic structure of conjugated systems. VI. Proc. R. Soc. Lond. Ser. A.

[29] Schmalz T.G., Cooper D.L. (2002). A valence bond view of fullerenes. Valence Bond Theory.

[30] Coulson C.A., Fisher I. (1949). Notes on the molecular orbital treatment of the hydrogen molecule. Phil. Mag..

[31] Goddard W.A. (1967). Improved quantum theory of many-electron systems. II. The basic method. Phys. Rev..

[32] Wannier G. (1937). The structure of electronic excitation levels in insulating crystals. Phys. Rev..

[33] Löwdin P.O. (1950). On the non-orthogonality problem connected with the use of atomic wave functions in the theory of molecules and crystals. J. Chem. Phys..

[34] Pople J.A. (1953). Electron interaction in unsaturated hydrocarbons. Trans. Faraday Soc..

[35] Pariser R., Parr R.G. (1953). A semi-empirical theory of the electronic spectra and electronic structure of complex unsaturated molecules. I. J. Chem. Phys..

[36] Pariser R., Parr R.G. (1953). A semi-empirical theory of the electronic spectra and electronic structure of complex unsaturated molecules. II. J. Chem. Phys..

[37] Fischer-Hjalmars I. (1965). Zero differential overlap in *π*-electron theories. Adv. Quantum Chem..

[38] Fischer-Hjalmars I. (1965). Deduction of the zero differential overlap approximation from an orthogonal atomic orbital basis. J. Chem. Phys..

[39] Fischer-Hjalmars I., Sinanoglu O. (1965). Orbital basis of zero differential overlap. Modern Quantum Chemistry.

[40] Slater J.C. (1951). Note on orthogonal atomic orbitals. J. Chem. Phys.

[41] McWeeny R.  (1954). The valence bond theory of molecular structure. I. Orbital theories and the valence-bond method. Proc. R. Soc. Lond. Ser. A.

[42] Malrieu J.P., Angeli C., Cimiraglia R. (2008). On the relative merits of non-orthogonal and orthogonal valence bond methods illustrated on the hydrogen molecule. J. Chem. Educ..

[43] Slater J.C. (1963). Quantum Theory of Molecules and Solids, vol. 1.

[44] Pilar F.L. (1968). Elementary Quantum Chemistry.

[45] Gallup G.A., Cooper D.L. (2002). A short history of VB theory. Valence Bond Theory.

[46] Ruedenberg K. (1962). The physical nature of the chemical bond. Rev. Mod. Phys..

[47] Edmiston C., Ruedenberg K. (1964). Chemical binding in the water molecule. J. Phys. Chem..

[48] Feinberg M.J., Ruedenberg K., Mehler E. (1970). The origin of binding and antibinding in the hydrogen molecule-ion. Adv. Quantum Chem..

[49] Feinberg M.J., Ruedenberg K. (1971). Paradoxical role of the kinetic-energy operator in the formation of the covalent bond. J. Chem. Phys..

[50] Feinberg M.J., Ruedenberg K. (1971). Heteropolar one-electron bond. J. Chem. Phys..

[51] Ruedenberg K., Daudel R. (1975). The nature of the chemical bond: An energetic view. Localization and Delocalization in Quantum Chemistry.

[52] Ruedenberg K., Schmidt M.W. (2007). Why does electron sharing lead to covalent bonding? A variational analysis. J. Comp. Chem..

[53] Ruedenberg K., Schmidt M.W. (2009). Physical understanding through variational reasoning: Electron sharing and covalent bonding. J. Phys. Chem. A.

[54] Bitter T., Ruedenberg K., Schwarz W.H.E. (2007). Towards a physical understanding of electron-sharing two-center bonds. I. General aspects. J. Comp. Chem..

[55] Bitter T., Wang S.G., Ruedenberg K., Schwarz W.H.E. (2010). Towards a physical understanding of electron-sharing two-center bonds. II. Pseudo-potential based analysis of diatomic molecules. Theor. Chem. Acc..

[56] Schmidt M.W., Ivanic J., Ruedenberg K. (2014). Covalent bonds are created by the drive of electron waves to lower their kinetic energy through expansion. J. Chem. Phys..

[57] Schmidt M.W., Ivanic J., Ruedenberg K., Frenking G., Shaik S. (2014). The physical origin of covalent bonding. The Chemical Bond. Fundamental Aspects of Chemical Bonding.

[58] Hubbard J. (1963). Electron correlation in narrow energy bands. Proc. R. Soc. Lond. Ser. A.

[59] Hubbard J. (1964). Electron correlation in narrow energy bands. III. An improved solution. Proc. R. Soc. Lond. Ser. A.

[60] Atchity G., Ruedenberg K. (1997). Determination of diabatic states through enforcement of configurational uniformity. Theor. Chem. Acc..

[61] Angeli C., Cimiraglia R., Malrieu J.P. (2013). Non-orthogonal and orthogonal valence bond wavefunctions in the hydrogen molecule: The diabatic view. Mol. Phys..

[62] Nakamura H., Truhlar D. (2001). The direct calculation of diabatic states based on configurational uniformity. J. Chem. Phys..

[63] Nakamura H., Truhlar D. (2002). Direct diabatization of electronic states by the fourfold way. II. Dynamical correlation and rearrangement processes. J. Chem. Phys..

[64] Nakamura H., Truhlar D. (2003). Extension of the fourfold way for calculation of global diabatic potential energy surfaces of complex, multiarrangement, non-Born-Oppenheimer systems: Application to HNCO (S_0_, S_1_). J. Chem. Phys..

[65] Kabbaj O.K., Volatron F., Malrieu J.P. (1988). A nearly diabatic description of S_*N*_2 reactions: The collinear ${\text{H}}_{3}^{+}$ model. Chem. Phys. Lett..

[66] Kabbaj O.K., Lepetit M.B., Malrieu J.P., Sini G., Hiberty P.C. (1991). S_*N*_2 reactions as Two-State Problems: Diabatic MO-CI Calculations on ${\text{Li}}_{3}^{-}$, Li_2_H^−^, ${\text{Cl}}_{3}^{-}$, and ClCH_3_Cl^−^. J. Am. Chem. Soc..

[67] Malrieu J.P., Guihery N., Calzado C.J., Angeli C. (2007). Bond electron pair: Its relevance and analysis from the quantum chemistry point of view. J. Comp. Chem..

[68] Lennard-Jones J. (1954). New ideas in chemistry. Adv. Sci..

[69] Scemama A., Caffarel M., Savin A. (2007). Maximum probability domains from quantum monte carlo calculations. J. Comp. Chem..

[70] Lüchow A. (2014). Maxima of |Ψ|^2^: A connection between quantum mechanics and Lewis structures. J. Comp. Chem..

[71] Schmidt M.W., Baldridge K.K., Boatz J.A., Elbert S.T., Gordon M.S., Jensen J.H., Koseki S., Matsunaga N., Nguyen K.A., Su S.J. (1993). General atomic and molecular electronic structure system. J. Comp. Chem..

